# Synthetic protein circuits for programmable control of mammalian cell death

**DOI:** 10.1016/j.cell.2024.03.031

**Published:** 2024-04-23

**Authors:** Shiyu Xia, Andrew C. Lu, Victoria Tobin, Kaiwen Luo, Lukas Moeller, D. Judy Shon, Rongrong Du, James M. Linton, Margaret Sui, Felix Horns, Michael B. Elowitz

**Affiliations:** 1 Division of Biology and Biological Engineering, California Institute of Technology, Pasadena, CA 91125, USA; 2 Howard Hughes Medical Institute, California Institute of Technology, Pasadena, CA 91125, USA; 3 UCLA-Caltech Medical Scientist Training Program, University of California, Los Angeles, CA 90095, USA; 4 UC Davis-Caltech Veterinary Scientist Training Program, University of California, Davis, CA 95616, USA; 5 Current address: Department of Medical Biochemistry and Biophysics, Karolinska Institutet, Stockholm 17165, Sweden

**Keywords:** Synthetic biology, synthetic circuit, protein circuit, cell death, synpoptosis, apoptosis, pyroptosis

## Abstract

Natural cell death pathways such as apoptosis and pyroptosis play dual roles: they eliminate harmful cells and modulate the immune system by dampening or stimulating inflammation. Synthetic protein circuits capable of triggering specific death programs in target cells could similarly remove harmful cells while appropriately modulating immune responses. However, cells actively influence their death modes in response to natural signals, making it challenging to control death modes. Here, we introduce naturally inspired “synpoptosis” circuits that proteolytically regulate engineered executioner proteins and mammalian cell death. These circuits direct cell death modes, respond to combinations of protease inputs, and selectively eliminate target cells. Furthermore, synpoptosis circuits can be transmitted intercellularly, offering a foundation for engineering synthetic killer cells that induce desired death programs in target cells without self-destruction. Together, these results lay the groundwork for programmable control of mammalian cell death.

## In brief

Naturally inspired synthetic “synpoptosis” protein circuits allow programmable control of cell death programs in mammalian cells.

## In Breif

Naturally inspired protein engineering produces synpoptosis circuits that programmably control user-selectable death programs in target mammalian cells.

## Graphical Abstract

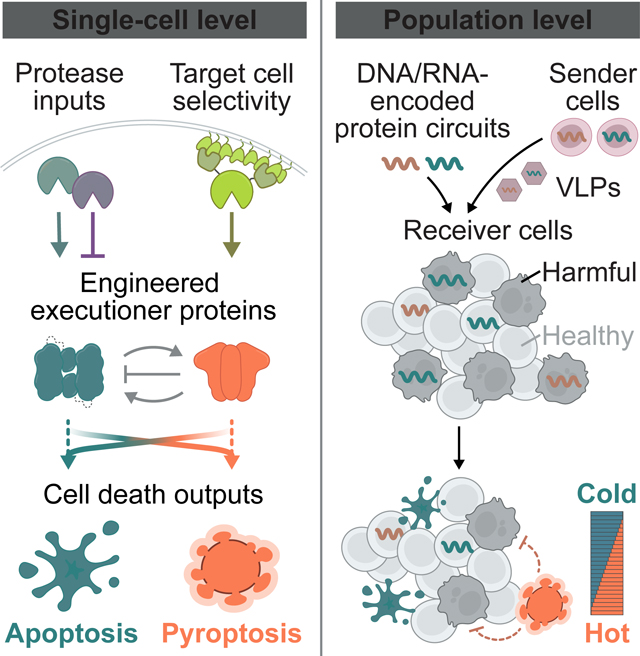

## INTRODUCTION

Mammalian systems use distinct cell death programs to eliminate harmful cells and shape immunity.^1–4^ Apoptosis is immunologically silent or “cold”.^5,6^ By contrast, pyroptosis is immunologically “hot” and involves substantial release of damage-associated molecular patterns (DAMPs).^7–18^

Apoptosis and pyroptosis can each be advantageous depending on immunological context. The immunostimulatory nature of pyroptosis can promote cell killing. For example, inducing pyroptosis in a 15% minority of cells was sufficient to clear an entire tumor by boosting anti-tumor immunity.^19^ Consistently, expression of the gasdermin (GSDM) family of pore-forming proteins, the executioners of pyroptosis, positively correlates with cancer patient survival, and cytotoxic lymphocytes upregulate GSDM expression in cancer cells.^20^ To escape pyroptosis, cancer cells generate loss-of-function GSDM mutations, silence GSDM expression, and express non-pyroptotic GSDM variants.^21–24^ Although beneficial in some contexts, pyroptosis can lead to pathological inflammation if triggered excessively.^25^ Therefore, it would be desirable to controllably induce apoptosis or pyroptosis and tune their relative frequencies.

Existing cell-killing approaches cannot fully direct the mode of cell death. Cytotoxic drugs are often limited to triggering apoptosis in cold tumors.^26,27^ CAR-T cells can effectively target cells expressing either a single antigen or multiple antigens.^28–31^ However, to kill target cells, CAR-T cells typically use granzymes, which may induce either apoptosis or pyroptosis.^7^ Granzyme-independent approaches have also been attempted, including engineered TRAIL-presenting cells^32^ and synthetic circuits that regulate caspases, BID, or BAX.^33–35^ However, these approaches are similarly restricted to induction of apoptosis.

To enable tailored control of cell death, we need a set of synthetic circuits with the following features. First, the circuits should allow activation and repression of both apoptosis and pyroptosis. Second, they should steer the mode of cell death in various cell contexts. Third, they should allow the integration and computation of multiple input signals. Fourth, they should be able to selectively kill target cells. Fifth, they should support cell-cell transmission, offering the potential to engineer synthetic killer cells that use designed death programs to eliminate other cells.

Here, we present synthetic protein-level cell death circuits, collectively termed “synpoptosis” circuits, that demonstrate the above features. To engineer these circuits, we took inspiration from natural cell death pathways that use regulated proteolysis along with protein-level caging and degradation mechanisms. Synpoptosis circuits provide a foundation for rationally designed, programmable control of mammalian cell death.

## RESULTS

### Synpoptosis circuits control user-selectable cell death programs

Due to the inherent tendency of cells towards apoptosis or pyroptosis, natural inducers do not completely direct the mode of cell death. For example, when cytotoxic lymphocytes deliver granzymes into target cells, target cells lacking GSDMs undergo apoptosis, whereas those expressing functional GSDMs undergo pyroptosis.^20,21^ This lack of control allows target cells to activate undesirable death programs that favor their own survival or trigger systemic toxicity.^7^

**Figure 1.**
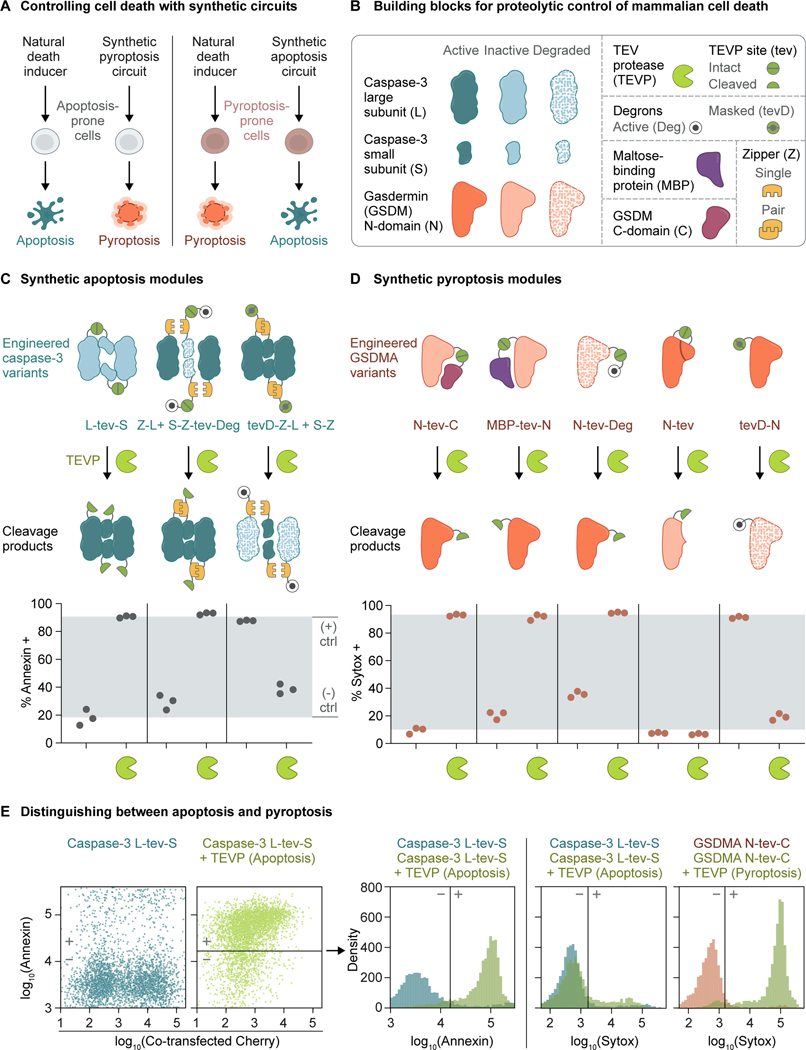
Synpoptosis circuits control user-selectable cell death programs **(A)** Synthetic cell death “synpoptosis” circuits steer the mode of cell death by operating orthogonally to cell-intrinsic death programs. **(B)** Molecular building blocks of synpoptosis circuits include caspase-3 subunits, gasdermin domains, viral proteases such as TEVP, degrons, maltose-binding protein, and leucine zippers. **(C)** Synthetic apoptosis modules use viral proteases, such as TEVP, to activate or repress engineered variants of caspase-3. We transiently transfected HEK cells with plasmid DNA encoding the synthetic modules and then quantified cell death by staining and flow cytometry. Throughout the figure, the gray window indicates the fractions established by negative and positive transient DNA transfection controls; dots represent biological replicates (distinct culture wells). **(D)** Synthetic pyroptosis modules similarly use TEVP to regulate engineered GSDMA. **(E)** Apoptosis and pyroptosis exhibit different staining patterns with Annexin and Sytox. Annexin stains both apoptotic and pyroptotic cells, while Sytox primarily stains pyroptotic cells. We calculated the fraction of cells that stained positive for each dye after gating on the transfected cells based on fluorescence of a co-transfected marker. Data represent three independent experiments. See also Figure S1.

To design synpoptosis circuits capable of steering the mode of cell death (Figure 1A), we drew inspiration from naturally occurring cell death programs. Caspases, as proteases, contribute to apoptosis by activating the caspase-activated DNase and the XKR8 phospholipid scramblase, among other pro-apoptotic factors.^36–39^ Caspases can also cleave GSDMs for pore formation in pyroptosis.^7,9^ Additionally, in synthetic biology, proteases represent a “common currency”, as they can be engineered to perform various signal-processing tasks.^40–44^ Therefore, proteolysis is a promising mode of synthetic regulation of cell death (Figure 1B–D).

In most experiments, we used human embryonic kidney 293 (HEK) cells because they express low levels of endogenous GSDMs, providing a clear background for synthetically induced cell death (Figure S1A). We transiently co-transfected HEK cells with DNA encoding the circuits, often along with Cherry, a fluorescent protein marker, and analyzed cells by flow cytometry 16 to 24 hours later. To quantify cell death, we stained the cells with Annexin, Sytox, or both. Annexin stains apoptotic cells by binding to phosphatidylserine exposed on the outer leaflet, and pyroptotic cells by binding to phosphatidylserine in the inner leaflet after membrane permeabilization.^45^ In contrast, Sytox, a membrane-impermeable dye, primarily stains pyroptotic cells.^46^ Therefore, apoptotic cells are typically Annexin-high and Sytox-low, while pyroptotic cells are Annexin-high and Sytox-high (Figure S1B). For simplicity, in experiments involving only a single death mode, we often used a single relevant dye. To focus on cells influenced by the circuits, we calculated the fraction of Annexin- or Sytox-positive cells after gating on the fluorescent co-transfection marker (Figure S1C, and see Methods).

Previous work showed that the tobacco etch virus protease (TEVP) could activate a modified caspase-3 whose natural cleavage site between its large and small subunits was replaced by a TEVP cleavage site.^33^ Using Annexin staining, we verified the ability of the modified caspase-3 to kill HEK cells when co-transfected with TEVP (Figure 1C, first module). Further, the cells only modestly took up Sytox, suggesting apoptosis (Figure 1E).

To enable more complex functions for control of apoptosis downstream, we engineered additional caspase-3 variants that can be modulated by TEVP cleavage. We first fused the large and small subunits of caspase-3 to heterodimerizing leucine zippers to produce a constitutively active non-covalent complex.^47^ Then, we appended a TEVP-removable dihydrofolate reductase degron to the small subunit. This degron is constitutively active,^48^ substantially reducing caspase-3 activity in the absence of TEVP. When TEVP was expressed, proteolytic removal of the degron efficiently activated apoptosis (Figure 1C, second module). To enable deactivation of apoptosis through proteolysis, we fused a TEVP site-caged N-degron^49,50^ to the large subunit of caspase-3. The caged N-degron is inactive because its N-terminal destabilizing residue, a tyrosine, is preceded by the TEVP site sequence. Upon TEVP cleavage, the destabilizing residue is exposed, activating the N-degron. As such, TEVP cleavage repressed apoptosis (Figure 1C, third module).

Next, we sought to engineer proteolytic control of pyroptosis. We inserted the cleavage sites of TEVP, tobacco vein mottling virus protease (TVMVP), and hepatitis C virus protease (HCVP) into a linker region between the N-terminal (pore-forming) and C-terminal (auto-inhibitory) domains of three mammalian GSDMs. Each engineered GSDM effectively triggered pyroptosis in HEK cells in the presence of the cognate protease, as shown by Sytox staining (Figure 1D, first module, Figure 1E, Figure S1D). We focused on GSDMA in subsequent experiments because it is thought to be orthogonal to endogenous host pathways.^51–53^

As with caspase-3, we designed additional GSDM variants that allow positive and negative control over pyroptosis. GSDM structures showed that the N-termini of GSDMs need to be accessible to lipids for pore formation.^54–58^ Therefore, we caged the activity of the GSDMA N-terminal domain by blocking its N-terminus with a bulky maltose-binding protein. Insertion of a TEVP cleavage site allowed proteolytic removal of the bulky tag to restore pore-forming activity (Figure 1D, second module). In a complementary approach, we fused a constitutively active degron to the GSDMA N-terminal domain to lower its activity. In this configuration, removal of the degron by TEVP also induced pyroptosis (Figure 1D, third module). To proteolytically switch off GSDM activity, we initially mimicked a natural mechanism in which the GSDMD N-terminal domain is inactivated through cleavage by caspase-3.^59,60^ However, inserting the TEVP cleavage site at an equivalent location within GSDMA abolished its activity (Figure 1D, fourth module). As an alternative strategy, we fused the GSDMA N-terminal domain to a TEVP site-caged N-degron. This design allowed protease-mediated suppression of pyroptosis (Figure 1D, fifth module). We note that cells also naturally use loop architectures to downregulate GSDM activity.^61–63^ These loops are at present challenging to construct synthetically, but motivate future synpoptosis circuit designs that fine-tune the penetrance of cell death.

Together, these results demonstrate that synthetic regulated proteolysis circuit modules can bidirectionally control apoptosis and pyroptosis.

### Synpoptosis circuits lead to canonical features of cell death

Cell death induced by synpoptosis circuits exhibited Annexin and Sytox staining patterns indicative of apoptosis and pyroptosis. To further characterize circuit-induced cell death, we looked for additional canonical features associated with naturally occurring cell death.

**Figure 2.**
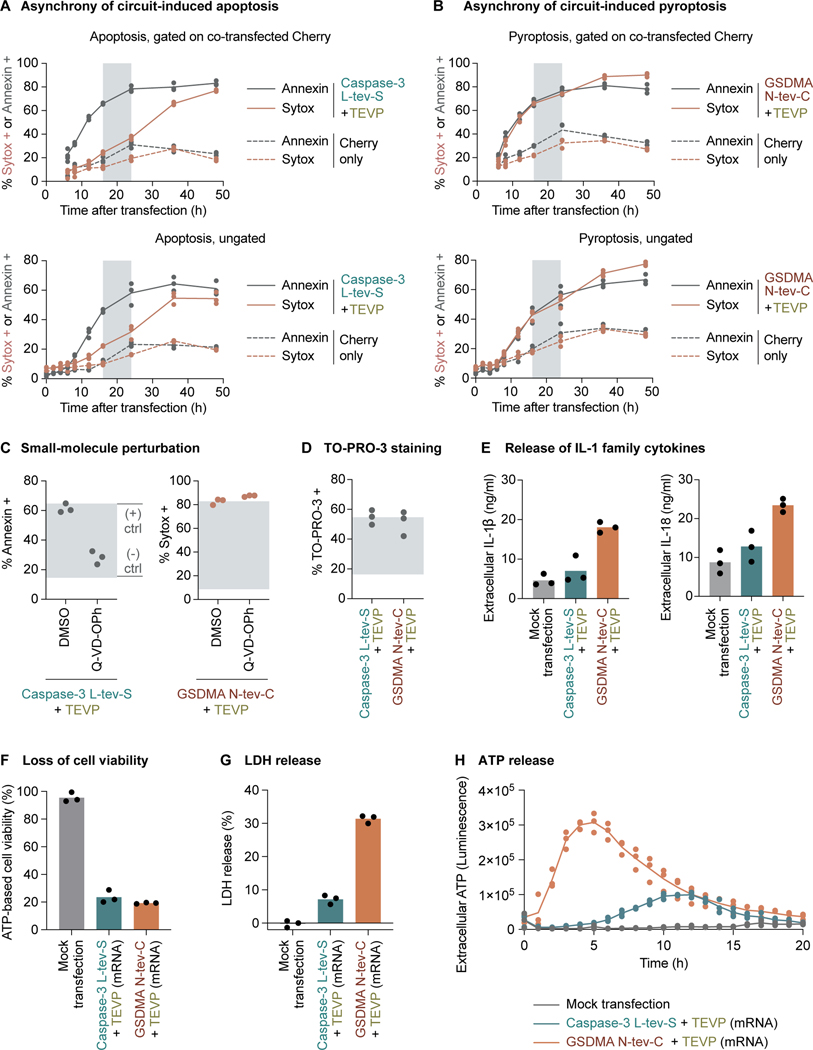
Synpoptosis circuits lead to canonical features of cell death **(A)** Transient transfection of HEK cells with plasmid DNA encoding the TEVP-activated caspase-3 circuit triggered asynchronous apoptosis, shown by flow cytometry. Between 16 and 24 hours after transfection (gray window), Sytox remains low in apoptotic cells and therefore can reliably distinguish between apoptotic and pyroptotic cells. Throughout the paper, dots represent biological replicates (distinct culture wells). **(B)** Similarly, the TEVP-activated GSDMA circuits triggered asynchronous pyroptosis. **(C)** Q-VD-OPh suppressed circuit-induced apoptosis but not pyroptosis, shown by flow cytometry. The gray window indicates the fractions established by negative and positive transient DNA transfection controls. **(D)** TO-PRO-3 stains cells killed by the apoptosis and pyroptosis circuits, shown by flow cytometry. **(E)** The apoptosis and pyroptosis circuits differently triggered the release of IL-1β and IL-18 from engineered HEK cells that stably express these cytokines. **(F)** Transient transfection using in vitro transcribed mRNA transcripts of the synpoptosis circuits led to loss of ATP-based cell viability. **(G)** The mRNA version of the pyroptosis circuit triggered more LDH release than the apoptosis circuit. **(H)** The mRNA version of the pyroptosis circuit triggered more ATP release than the apoptosis circuit. The same wells were repeatedly captured across a time course. See also Figure S2.

Drugs and death ligands typically induce cell death asynchronously within a cell population.^64–66^ To assess whether synpoptosis is similarly asynchronous, we transfected HEK cells with TEVP-activated caspase-3 and GSDMA circuits, and then double-stained the cells with Annexin and Sytox for flow cytometry analysis over a time span. Both synthetic apoptosis and pyroptosis exhibited asynchrony (Figure 2A and B). In the case of apoptosis, Annexin-positivity preceded Sytox-positivity, indicating a sequence of apoptosis followed by eventual cell lysis (Figure 2A). In contrast, the pyroptotic cells displayed a simultaneous increase in Annexin and Sytox signals (Figure 2B). Similar dynamic patterns were observed without gating on the co-transfected Cherry, demonstrating robust circuit effects on a population level (Figure 2A and B). Because the apoptotic cells eventually progress to cell lysis, we confined our flow cytometry analysis to a window between 16 and 24 hours post-transfection to distinguish between apoptosis and pyroptosis (Figure S1B).

Next, considering that naturally occurring cell death is influenced by the concentration of executioner molecules,^67^ we investigated whether synpoptosis is concentration-sensitive. Indeed, engineered auto-inhibited caspase-3 and GSDMA, when expressed at high levels indicated by co-transfected Cherry, induced noticeable cell death even without the activating TEVP (Figure S2A). Further, titrating the amount of plasmid DNA encoding the synpoptosis circuits enabled DNA dose-dependent control over killing fractions (Figure S2B). At a constant amount of plasmid DNA, tuning could achieved by adjusting mRNA dosage (Figure S2C) through a synthetic miRNA-based incoherent feedforward loop (IFFL) motif,^68,69^ which allows DNA dosage-independent control of protein expression.^70,71^

Then, because naturally occurring cell death can be modulated by small-molecule compounds, we investigated the effect of Q-VD-OPh, a caspase inhibitor, on synpoptosis circuits (Figure 2C). In line with expectations, Q-VD-OPh markedly attenuated apoptosis induced by the caspase-3 circuit, but did not affect pyroptosis mediated by the GSDMA circuit. We also asked whether cells treated with synpoptosis circuits stain positive for TO-PRO-3, a dye that enters apoptotic cells through pannexin channels and pyroptotic cells through permeabilized membranes. As anticipated, cells treated with either circuit showed positive staining for TO-PRO-3 (Figure 2D).

A physiologically important feature of pyroptosis is the release of pro-inflammatory cytokines, notably the interleukin (IL)-1 family including IL-1β and IL-18.^72–74^ For demonstration purposes, we generated a HEK cell line that stably expresses IL-1β and IL-18. We observed an obvious increase in supernatant levels of IL-1β and IL-18 in cells treated with the GSDMA circuit, compared to cells treated with the caspase-3 circuit or the mock transfection control (Figure 2E).

Furthermore, we examined the morphological characteristics of cells treated with synpoptosis circuits (Figure S2D). We analyzed cell shapes by phase-contrast microscopy. Mock-transfected cells displayed a mostly flat and extended appearance. In contrast, cells transfected with the caspase-3 circuit showed extensive grape-like membrane blebbing and cell shrinkage, characteristic of apoptosis. On the other hand, cells transfected with the GSDMA circuit showed cell rounding and parachute-like membrane swelling, indicative of pyroptosis. Additionally, the nuclear localized H2B-Cherry marker allowed us to visualize nuclear fragmentation in cells treated with the caspase-3 circuit.

Lastly, mRNA represents a rapidly growing modality for transient therapeutic protein expression.^75,76^ To determine whether the mRNA versions of the synpoptosis circuits could generate the expected killing effects, we generated mRNA by in vitro transcription, used it to transfect cells, and then read out three indicators of cell death. A luciferase-based assay to quantify ATP levels in the cell culture, an indicator of viable cells, revealed that mRNA-encoded synpoptosis circuits metabolically inactivated cells after killing (Figure 2F). The GSDMA circuit resulted in substantial release of lactate dehydrogenase (LDH), a pyroptotic signature (Figure 2G), as well as adenosine triphosphate (ATP), a small-molecule DAMP (Figure 2H).

Together, these results demonstrate that synpoptosis circuits induce canonical features of cell death programs and function expectedly whether delivered as DNA or mRNA.

### Synpoptosis circuits direct the mode of cell death

Instead of leaving the choice of death mode up to cells, we sought to use synpoptosis circuits to actively direct cell death. Specifically, we aimed to drive pyroptosis in apoptosis-prone cells, promote apoptosis in pyroptosis-prone cells, and trigger either death program in cells capable of both apoptosis and pyroptosis. Because these experiments involve mixed programs, we double-stained the cells with Sytox and Annexin to distinguish between apoptosis and pyroptosis. Given that Annexin labels both apoptotic and pyroptotic cells, we used it as a proxy for total cell death.

**Figure 3.**
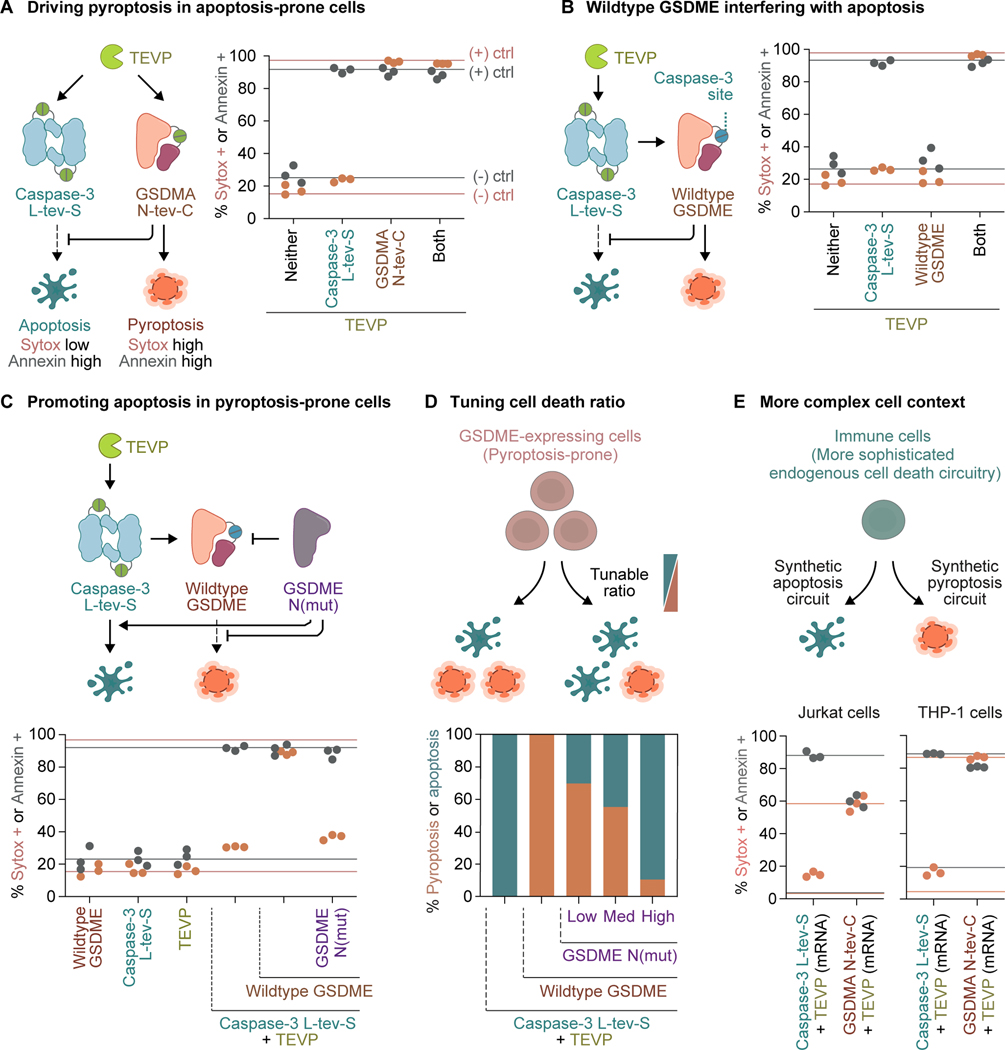
Synpoptosis circuits direct the mode of cell death **(A)** Transient transfection of plasmid DNA encoding TEVP and engineered TEVP-activatable caspase-3 induced apoptosis in HEK cells, shown by flow cytometry. TEVP-activatable GSDMA overrode the apoptotic program, leading to pyroptosis. Throughout the figure, horizontal lines indicate the fractions established by negative and positive transient DNA transfection controls, established separately for Annexin and Sytox; dots represent biological replicates (distinct culture wells). **(B)** Cells transfected with plasmid DNA encoding wildtype GSDME, a natural substrate of caspase-3, underwent pyroptosis in response to TEVP-mediated caspase-3 activation. **(C)** GSDME N(mut) overcame the tendency of wildtype GSDME-expressing cells to undergo pyroptosis downstream of caspase-3 activation and promoted apoptosis. **(D)** The ratio between the two forms of cell death is tunable by adjusting the plasmid DNA amount of GSDME N(mut) relative to wildtype GSDME (low, 1:1; medium, 2:1; high, 4:1). The heights of the stacked bars indicate means of three biological replicates (distinct culture wells) measured by flow cytometry. **(E)** In vitro transcribed mRNA versions of synpoptosis circuits drove apoptosis and pyroptosis in Jurkat and THP-1 cells, which have more sophisticated endogenous cell death circuitry than HEK cells. See also Figure S3.

To drive pyroptosis in apoptosis-prone cells, we established a synthetic system that mimics a GSDM-negative cell context, with TEVP activating caspase-3 to induce apoptosis. In this background, ectopically expressing TEVP-activatable GSDMA converted apoptosis to pyroptosis (Figure 3A), suggesting that GSDMA dominated over caspase-3. While this dominance provided a straightforward approach to drive pyroptosis in GSDM-negative cells, it could be exploited by cells seeking to evade apoptosis. Indeed, cells that express GSDME, a natural substrate for caspase-3, undergo pyroptosis downstream of caspase-3 activation.^26,27^ This effect can be recapitulated by ectopically expressing wildtype GSDME, which alone did not induce cell death, but caused pyroptosis in response to TEVP-mediated caspase-3 activation (Figure 3B).

To promote apoptosis in cells expressing GSDME, we searched for a protein-level GSDME inhibitor. Recent studies revealed that GSDMB has alternatively spliced variants, with the non-pyroptotic variants inhibiting the pyroptotic ones.^23,24^ Motivated by this trans inhibition mechanism, we tested a panel of GSDME N-terminal domain mutants, including some associated with cancer,^21^ and identified I217N as defective in inducing pyroptosis and capable of inhibiting the wildtype counterpart (Figure S3A). Then, we co-transfected this mutant along with wildtype GSDME, TEVP, and TEVP-activatable caspase-3. The mutant expectedly suppressed pyroptosis, as evidenced by reduced Sytox uptake, while permitting caspase-3-induced apoptosis, as read out by high Annexin levels (Figure 3C).

The results above demonstrate that synpoptosis circuits can guide cells towards apoptosis or pyroptosis irrespective of their intrinsic preferences. However, to leverage the pro-inflammatory benefits of pyroptosis without triggering excessive inflammation, or conversely, the immunosuppressive benefits of apoptosis without curbing beneficial inflammation, it would be ideal to be able to adjust the relative levels between the two modes of cell death. By titrating down the GSDME inhibitor, we could attenuate pyroptosis to various degrees while still allowing activated caspase-3 to induce apoptosis (Figure S3B). Further quantification using the Annexin signal as a measure of total cell death and the Sytox signal as an indicator of pyroptosis revealed the tunable ratio between the two death programs (Figure 3D).

HEK cells are pyroptosis-incompetent without ectopically introduced GSDMs. To assess the efficacy of synpoptosis circuits in cells with more sophisticated endogenous death circuitry, we selected two widely used immune cell lines, Jurkat and THP-1. We transfected Jurkat and THP-1 cells with mRNA encoding TEVP-activated caspase-3 and GSDMA circuits and found that the cells died through the anticipated programs (Figure 3E). Further, to validate the orthogonality of synpoptosis circuits to endogenous death circuitry, we tested the circuits in GSDMD-knockout (KO) THP-1 cells. As expected, the circuits killed the GSDMD-KO THP-1 cells similarly to the wildtype cells (Figure S3C).

Together, these results demonstrate that synpoptosis circuits can direct the mode of cell death in various cell contexts and tune the ratios between apoptosis and pyroptosis.

### Synpoptosis circuits perform combinatorial computation

While engineered executioners enable control of cell death modes, taking advantage of upstream inputs would allow synpoptosis circuits to target specific cells. Perhaps for similar reasons, natural cell death pathways respond to logical combinations of inputs. For instance, either caspase-1 or caspase-11 activates GSDMD, functioning as an OR-like gate.^77^ Both GSDMD cleavage and its lipidation are necessary for pyroptosis, forming an AND-like gate.^78,79^

The activation and repression synpoptosis modules developed above can be combined to achieve such combinatorial logic gating functions. We focused on three biologically relevant gates: triggering cell death in the presence of Inputs 1 AND 2; in the presence of Input 1 OR 2; and in the presence of Input 1 AND the absence of Input 2. While the first and third gates increase specificity by killing cells with the two inputs in the right combinations, the second gate broadens specificity and could mitigate antigen escape.^80^

**Figure 4.**
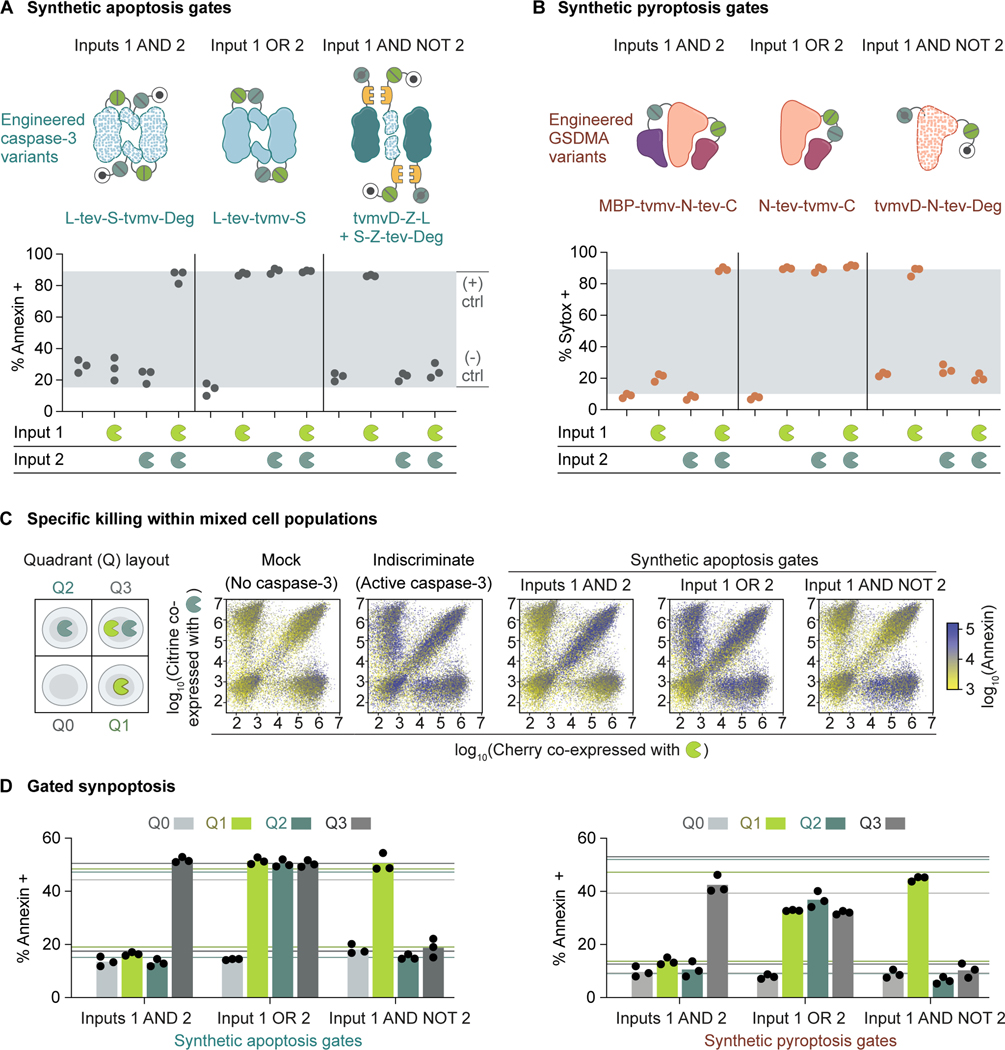
Synpoptosis circuits perform combinatorial computation **(A)** Synpoptosis circuits enabled context-dependent apoptosis by responding to logical combinations of protease inputs (TEVP as Input 1 and TVMVP as Input 2). In the first demonstration, transient transfection of plasmid DNA encoding both protease inputs must be present to activate the engineered caspase-3 and cause apoptosis, shown by flow cytometry. In the second demonstration, either input is sufficient to trigger apoptosis. In the third demonstration, apoptosis occurs only when the first input is present and the second input is absent. Throughout the figure, the gray window indicates the fractions established by negative and positive transient DNA transfection controls; dots represent biological replicates (distinct culture wells). **(B)** Similar design principles were applied to engineer the same gating functions for pyroptosis. **(C)** Using these synpoptosis gates, we can eliminate specific cells within mixed populations that exhibit distinct intracellular states defined by the expression profiles of input proteases and their fluorescence reporters, shown by flow cytometry. The cell states were established by stable protein expression using lentiviral transduction. Data represent three independent experiments. **(D)** Quantification of Annexin staining revealed the efficacy of the synpoptosis gates. Horizontal lines indicate quadrant-specific negative and positive controls of the Annexin signal. Bars indicate means of three biological replicates (distinct culture wells). See also Figure S4.

To trigger apoptosis in the presence of Inputs 1 (TEVP) AND 2 (TVMVP), we fused TEVP-activatable caspase-3 to a TVMVP-removable degron (Figure 4A, Inputs 1 AND 2). In this design, Input 1 releases the linker constraint between the large and small subunits of the engineered caspase-3, while Input 2 protects the caspase-3 from degradation. By contrast, to trigger apoptosis in the presence of Input 1 OR 2, we placed tandem TEVP and TVMVP cleavage sites at the inter-subunit linker of caspase-3 (Figure 4A, Input 1 OR 2). In this configuration, either protease is sufficient to activate the engineered caspase-3. Similar principles can be used to trigger apoptosis in the presence of Input 1 AND the absence of Input 2, by adding a TEVP-removable degron to the small subunit and a TVMVP-activatable N-degron to the large subunit (Figure 4A, Input 1 AND NOT 2). By extension, using different configurations of degrons and cleavage sites, we constructed synthetic apoptosis executioners that perform more binary logic operations (Figure S4A). With some modifications, the gate designs were transferable to pyroptosis programs (Figure 4B and S4B).

As a proof of concept for combinatorial targeting, we tested the synpoptosis gates in a co-culture of engineered HEK cells that stably express Input 1, Input 2, or both, and wildtype cells that express neither. We co-expressed Input 1 with Cherry, and Input 2 with Citrine, such that the two fluorescent protein profiles represented input patterns (Figure 4C, Quadrant layout). We then transfected the co-culture of the four cell lines with the synthetic apoptosis gates to analyze their killing specificity. We used transfections of no caspase-3 (Figure 4C, Mock) and active caspase-3 (Figure 4C, Indiscriminate) to establish background and ceiling apoptosis levels, respectively. Transfection of the AND-gated caspase-3 triggered apoptosis predominantly in double-positive cells, suggesting specific killing of cells exhibiting both inputs (Figure 4C and D, Inputs 1 AND 2). By contrast, the OR-gated caspase-3 caused apoptosis in single-positive and double-positive cells, sparing only double-negative cells (Figure 4C and D, Input 1 OR 2). The caspase-3 variant designed to activate in the presence of Input 1 and the absence of Input 2 restricted apoptosis to the corresponding cells, as expected (Figure 4C and D, Input 1 AND NOT 2). These results are qualitatively consistent with experiments performed in a non-co-culture context (Figure 4A). Similar killing specificities were observed when we transfected the synthetic pyroptosis gates into the co-culture (Figure 4D).

Together, these results demonstrate that synpoptosis circuits can respond to combinatorial protease inputs and eliminate specific cells among mixed populations through logic gating.

### Synpoptosis circuits selectively eliminate target cells

A major motivation for engineering input-responsive synpoptosis circuits is to achieve target cell selectivity. Tissues generally contain heterogeneous mixtures of healthy and harmful cells. To protect healthy cells, we engineered synpoptosis circuits that selectively eliminate harmful cells by conditional circuit activation.

**Figure 5.**
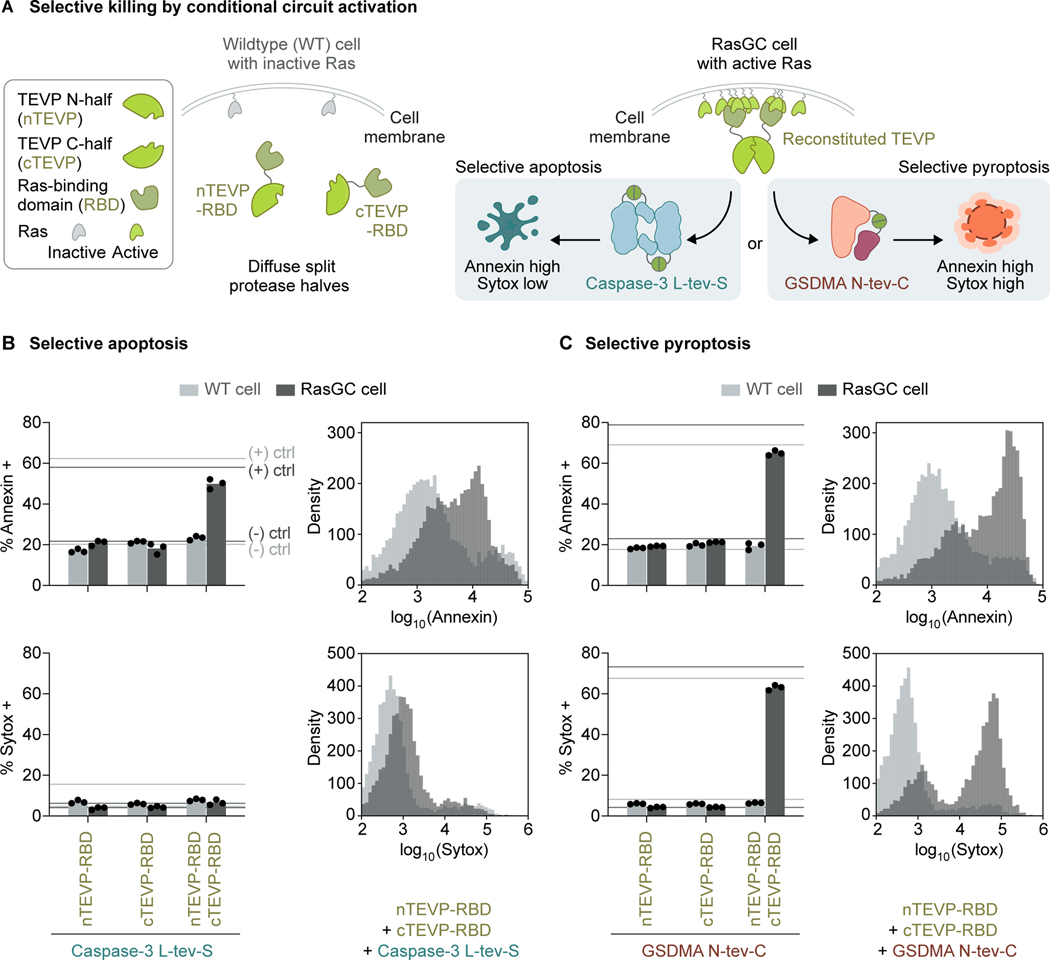
Synpoptosis circuits selectively eliminate target cells **(A)** Synpoptosis circuits can selectively kill target cells by incorporating a sensor module that identifies the target cells. We used a synthetic sensor of active Ras. The sensor is a split TEVP with each half – nTEVP and cTEVP – tethered to a Ras-binding domain (RBD). In wildtype (WT) HEK cells with inactive Ras, the sensor is catalytically inactive. In cells stably expressing active Ras (RasGC cells), TEVP is reconstituted by proximity. **(B)** Transient DNA transfection of a caspase-3-based synpoptosis circuit including the synthetic Ras sensor module enabled selective apoptosis of RasGC cells, but not WT cells. Throughout the figure, horizontal lines in the bar plots indicate the fractions established by negative and positive transient DNA transfection controls, separately for each dye and each cell line; dots represent biological replicates (distinct culture wells); bars indicate means; histograms represent three independent experiments. **(C)** Similarly, a Ras-sensing synpoptosis circuit using GSDMA as the output triggered selective pyroptosis of RasGC cells. See also Figure S5.

As a demonstration for target cell selectivity, we incorporated into synpoptosis circuits a synthetic sensor of Ras oncogene activity. The sensor consists of the inactive N-terminal and C-terminal halves of a split TEVP, with each half fused to a Ras-binding domain.^43^ Active Ras clusters at the membrane and binds the modified TEVP halves,^81,82^ reconstituting TEVP by proximity (Figure 5A). To evaluate the effectiveness of the Ras sensor, we tested it in HEK cells stably expressing the constitutively active H-Ras G12V mutant and a Cerulean fluorescent marker (hereafter, “RasGC cells”), along with a destabilized Citrine reporter that can be rescued by TEVP cleavage (Figure S5A). As a control, we analyzed a similar Citrine reporter cell line without the active Ras mutant (hereafter, “wildtype (WT) cells”). When we transiently transfected the sensor into the two cell lines, Citrine fluorescence increased in the RasGC cells more than the WT cells (Figure S5B and C), suggesting that the sensor can classify Ras activity states.

Next, we integrated the sensor into synpoptosis circuits for selective killing of RasGC cells. We co-transfected a circuit consisting of the sensor and TEVP-activatable caspase-3 into RasGC and WT cells (Figure 5B). According to Annexin staining, the RasGC cells underwent apoptosis to a degree similar to the positive control, a constitutively active apoptosis circuit containing full-length TEVP and TEVP-activatable caspase-3. The RasGC cells remained Sytox-low, indicating apoptosis rather than pyroptosis. Likewise, in synthetic pyroptosis experiments using TEVP-activatable GSDMA as the executioner instead of caspase-3, pyroptosis primarily occurred in the RasGC cells, shown by double positivity in Annexin and Sytox staining (Figure 5C). In both apoptosis and pyroptosis experiments, WT cells maintained low levels of Annexin and Sytox staining, suggesting selective killing of RasGC cells.

Together, these experiments demonstrate the ability of synpoptosis circuits to selectively kill target cells and spare non-target ones by interfacing with an endogenous intracellular signal.

### Synpoptosis circuits support intercellular operations

Thus far, we have demonstrated synpoptosis circuits through transient transfection. A nascent, alternative approach is cell-based delivery.^83–89^ In this paradigm, engineered sender cells release virus-like particles (VLPs) containing cargo that can be internalized by non-engineered receiver cells. This approach could enable the development of a synthetic killer cell that secretes VLPs expressing synpoptosis circuits. The circuits could then trigger user-selectable death programs in receiver cells, providing control over the mode of cell death.

**Figure 6.**
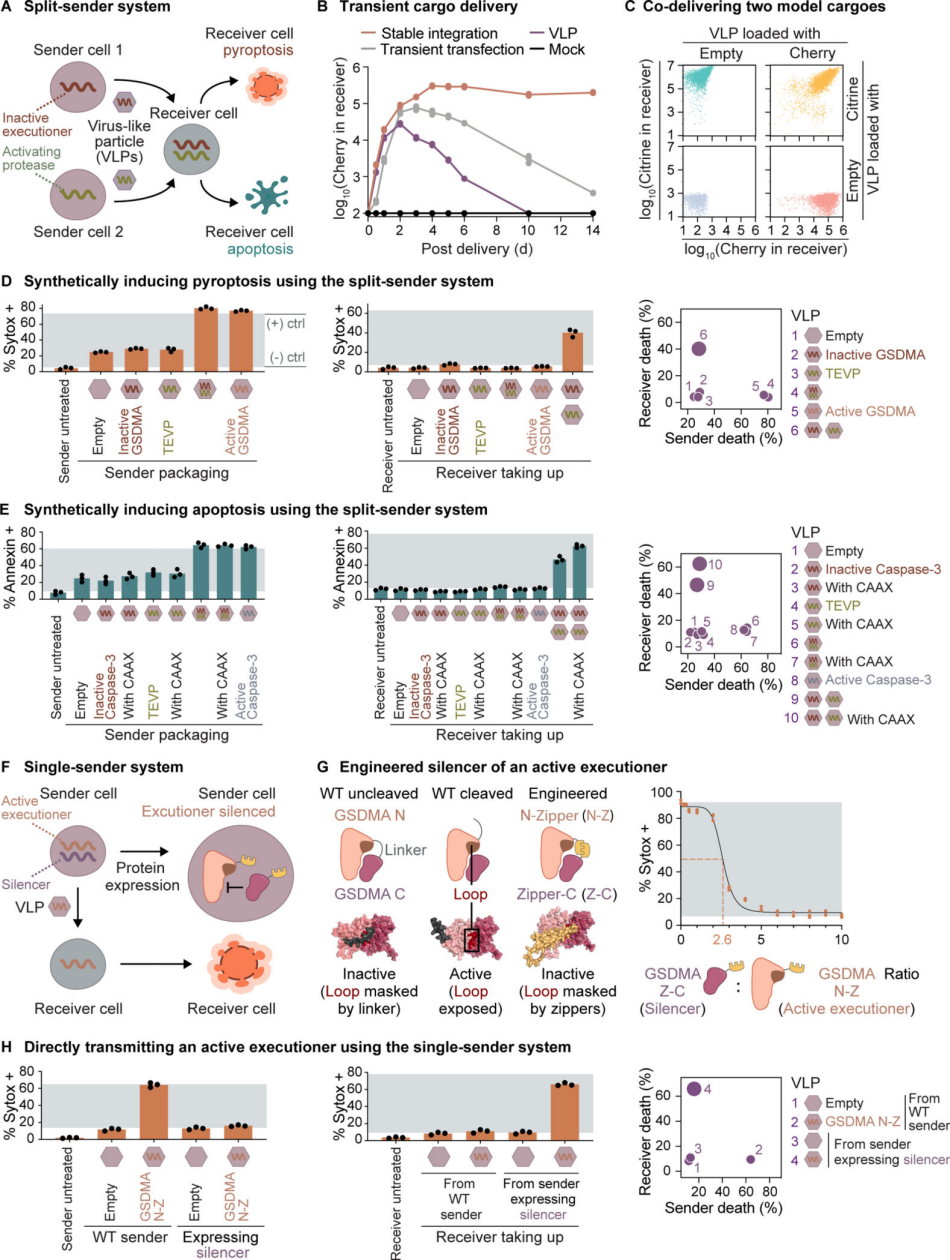
Synpoptosis circuits support intercellular operations **(A)** Synpoptosis circuits can be transmitted intercellularly by virus-like particles (VLPs). To address the key challenge of sender cell death, a simple strategy is to use a split-sender system, in which an inactive executioner and an activating protease are packaged separately. **(B)** A model VLP transiently delivered nucleic acid encoding Cherry from sender HEK cells to receiver HEK cells, where the cargo was expressed. Experiments were performed by supernatant transfer from sender to receiver cells after transient DNA transfection of sender cells. Line segments connect means at different time points. Dots represent biological replicates (distinct culture wells). **(C)** Two model cargoes, Cherry and Citrine, were separately packaged by two sender populations and co-delivered to the same receiver population, supporting the split-sender system. Scatter plots represent three independent experiments. **(D)** A synthetic pyroptosis circuit, consisting of inactive GSDMA and the activating protease TEVP, was delivered by VLP using the split-sender system. Throughout the figure, the gray window indicates the fractions established by negative and positive transient DNA transfection controls; bars indicate means; dots in the two-dimensional sender death-receiver death plots indicate means of three biological replicates (distinct culture wells). **(E)** Analogously, the split-sender system enabled VLP delivery of a synthetic apoptosis circuit, by separately packaging inactive caspase-3 and the activating protease TEVP. Appending CAAX tails to both circuit components enhanced apoptosis. **(F)** A more elegant, compact, single-sender system directly delivers an active executioner. This strategy requires sender-specific silencing of the active executioner. **(G)** A synthetic protein-level silencer (GSDMA Z-C) potently inhibited an engineered active executioner (GSDMA N-Z), likely by using leucine zippers (Z) to mask a loop critical for the executioner’s activity, shown by AlphaFold models. The inhibitory curve was obtained by transient transfection of HEK cells with plasmid DNA encoding the active executioner and the silencer at indicated plasmid mass ratios. **(H)** Senders transfected with plasmid DNA encoding the silencer, but not wildtype senders, could directly package VLPs that express the active executioner. See also Figure S6.

To deliver synpoptosis circuits with VLPs (Figure 6A), we first tested whether VLPs could enable high expression of nucleic acid-encoded model protein cargoes, Cherry and Citrine. As a model VLP, we used integration-deficient lentiviruses.^83^ We used HEK cells as senders to generate VLPs expressing Cherry. Supernatant containing the secreted VLPs led to transient expression of Cherry in receiver HEK cells (Figure 6B). Furthermore, separate VLPs encoding Cherry and Citrine could be co-delivered to the same receiver cells without cross-interference (Figure 6C).

We next asked whether we could transmit synpoptosis circuits intercellularly using VLPs. The key challenge is achieving high expression of the circuits in receivers without triggering cell death in senders. A simple way to avoid sender cell death is through an intersectional strategy, splitting a circuit into two components packaged by two separate senders, which we term a split-sender system (Figure 6A). As expected, when we split a pyroptosis circuit into TEVP and TEVP-activatable GSDMA, neither circuit component alone triggered substantial sender cell death. On the receiver side, HEK cells that took up separately packaged VLPs expressing both circuit components underwent pyroptosis, whereas cells that received VLPs loaded with only one cargo or empty VLPs survived (Figure 6D). The same split-sender system could also induce apoptosis (Figure 6E). To boost apoptosis efficiency, we enforced local proximity of the circuit components by appending a CAAX membrane-localization sequence to each.

The split-sender system described above demonstrates the capability of intercellularly transmitted synpoptosis circuits. However, it requires two sender populations that are toxic to each other, as VLPs produced by one population complete the death circuit in the other (Figure S6A). Engineering a compact single-sender system necessitates blocking the activity of synpoptosis circuits in sender cells without interfering with their activity in receivers (Figure 6F). Small-molecule inhibitors exist for caspases and GSDMD, but it is difficult to restrict their activity to sender cells. An alternative strategy is to attach conditional degrons that are active only in sender cells to the circuit components. However, degrons incorporated in GSDMA and caspase-3 could not fully inhibit cell death caused by either protein (Figure 1C and D).

To overcome these challenges, we engineered a GSDMA N-terminal domain variant, whose activity in senders could be caged by a separately expressed C-terminal domain variant (Figure 6G). We exploited the fact that in wildtype uncleaved GSDMA, the C-terminal domain folds back onto the N-terminal domain to sterically repress its pyroptotic activity by masking a loop that mediates lipid binding.^57^ Upon cleavage of the inter-domain linker, inhibition of GSDMA N-terminal domain is relieved because the cleaved linker no longer sterically masks the loop. Inspired by these natural interactions, we fused the N- and C-terminal domains to heterodimerizing leucine zippers, which should form an artificial linker-like motif that masks the lipid-binding loop like the natural uncleaved linker. Indeed, the zipper-attached C-terminal domain (silencer) markedly suppressed pyroptosis induced by the zipper-attached N-terminal domain (active executioner) (Figure S6B–D). As expected, the silencer did not inhibit pyroptosis induced by wildtype GSDMA N-terminal domain (Figure S6B). Finally, we specifically suppressed sender cell death by expressing the silencer in senders. The silencer-expressing senders did not undergo appreciable rates of pyroptosis when internally producing, or treated with externally produced, active executioner VLPs (Figure 6H and S6C). By contrast, non-engineered receivers taking up the VLPs underwent pyroptosis, whether the senders and receivers were separately cultured (Figure 6H) or co-cultured (Figure S6E).

Together, these results demonstrate that synpoptosis circuits provide a foundation for engineering synthetic killer cells that eliminate other cells without killing themselves.

## DISCUSSION

Mammalian systems trigger specific cell death programs depending on cell state and desired immunological outcome. Similarly, many therapeutic challenges could be addressed if one could kill the right cells in the right way.^75,76^ For example, synpoptosis circuits could be delivered to tumors to conditionally induce cancer cell death, in a manner that safely amplifies anti-tumor immunity. The circuits could also be used to eliminate cells playing other harmful roles, such as senescent cells, fibrotic cells, self-targeting immune cells, and infected host cells, and could do so with appropriate immune stimulation.

The utility of cell killing has been long recognized. Previous efforts mainly used death ligands,^32^ chemogenetics,^90,91^ such as inducible expression and chemically induced dimerization of executioners, and, more recently, optogenetics,^92–94^ which allows for spatiotemporal flexibility and higher-order assembly of executioners. While these methods generally enable cell death induction, they face several challenges: they have limited ability to direct the mode of cell death in various cell contexts; they largely cannot respond to combinatorial inputs and endogenous intracellular signals; and, they do not support cell-cell transmission, which is crucial for engineering synthetic killer cells that eliminate other cells without harming themselves.

To address these challenges, we engineered synpoptosis circuits using several design principles. First, proteolytic removal of inhibitory domains activates executioners (Figure 1 and 2). This principle is also used by nature, as both caspases and GSDMs are cleavage-activated proteins. Second, and conversely, cleavage-activated degrons allow proteases to suppress executioners (Figure 1). This suppression is useful in situations where inputs from healthy cells should deactivate cell death. Third, the two mechanisms above can be combined to allow for combinatorial control, which enables targeted death induction in specific cells (Figure 4). Fourth, mutant executioners can inhibit the activity of their wildtype counterpart (Figure 3).

In natural contexts, mammals use mixed cell death programs to modulate immunity.^23^ By contrast, traditional methods of inducing cell death either lead to only a single death mode, or passively delegate the choice of death mode to the target cell. This lack of active control poses a problem because the target cell often selects an undesirable death mode. Synpoptosis circuits offer active control by directing cell death independently of endogenous death programs and by adjusting the ratio between apoptosis and pyroptosis (Figure 3).

Endogenous intracellular signals can interact with engineered proteases, a common currency in synthetic protein circuits.^33,34^ Engineered executioners can in turn interface with such protease inputs, alone or in combination (Figure 4 and 5). As a proof of principle for target cell-selective killing, we incorporated a Ras sensor as an input to synpoptosis circuits (Figure 5). Looking ahead, we envision activating synpoptosis circuits using combinations of natural pathway activities and inputs from synthetic receptors like synNotch.^95,96^ Therefore, we anticipate that synpoptosis circuits will serve key roles in various synthetic biology applications.^97–99^

From a translational standpoint, delivery challenges have limited the advancement of synthetic circuits. A potential paradigm to improve delivery is the engineering of sender cells that navigate the body, home to disease sites, and selectively transmit synthetic circuits into receiver cells. We demonstrated the abilities to rationally program synpoptosis circuits, deliver them from one cell to another, and have them trigger customizable death programs in receiver cells (Figure 6). In the future, synthetic killer cells may additionally provide the ability to evade exhaustion, as they would not be affected by immunosuppression that limits natural cytotoxic lymphocytes. It will be exciting to see how synpoptosis circuits facilitate programmable control of cell death in various contexts, including cancer, senescence, fibrosis, autoimmunity, and infection.

### Limitations of the study

Synpoptosis circuits allow programmable control of apoptosis and pyroptosis but not other forms of cell death. The circuits are concentration-dependent and therefore may not be fully effective at minimal doses or in cells with low ectopic protein expression. The results here are limited to immortalized cell lines and do not demonstrate the performance of synpoptosis circuits in native cell contexts. Because Sytox also stains apoptotic cells that undergo necrosis, it is not fully specific to pyroptosis. To mitigate this issue, we performed analysis before substantial necrosis of apoptotic cells. Analysis of mixed induction of apoptosis and pyroptosis assumed exclusive outcomes; however, other hybrid death programs may exist. The study does not reveal the dynamics of synpoptosis circuit expression and activity in individual cells, which could be informative about the cell-cell variability of circuit effects. Finally, a remaining intriguing question is whether synpoptosis circuits could function in bacteria and other non-mammalian systems.

## STAR★METHODS

### RESOURCE AVAILABILITY

#### Lead contact

Further information and requests for resources and reagents should be directed to and will be fulfilled by the lead contact, Michael B. Elowitz (melowitz@caltech.edu).

#### Materials availability

Reagents used and generated in this study are available from the lead contact upon request, subject to the completion of a materials transfer agreement.

#### Data and code availability

- Raw and analyzed data associated with this paper have been deposited at CaltechDATA (data.caltech.edu) and will also be shared by the lead contact upon request. The link to the CaltechDATA page is listed in the key resources table.
- Original codes used for processing data and plotting figures have been deposited at GitHub and are publicly available as of the date of publication. The link to the GitHub page is listed in the key resources table.
- Any additional information required to reanalyze the data reported in this paper is available from the lead contact upon request.

### EXPERIMENTAL MODEL AND STUDY PARTICIPANT DETAILS

#### Tissue culture and cell lines

All cells were cultured under standard conditions, on tissue culture grade plastic plates and dishes (Thermo Fisher) or flasks (CELLTREAT), uncoated or coated with poly-D-lysine (Thermo Fisher), at 37 °C with 5% CO_2_ in humidified incubators (CellXpert C170i, Eppendorf). All cells were handled in sterile safety cabinets (SterilGARD III Advance or SterilGARD e3, Baker), and counted using the Countess 3 automated cell counter (Thermo Fisher). Dead cells were calculated using trypan blue (Invitrogen). Human embryonic kidney (HEK) cells (HEK293, T-REx-293, HEK293T, and HEK293FT variants), wildtype or engineered, were maintained in Dulbecco’s Modified Eagle Media (Themo Fisher) supplemented with 10% fetal bovine serum (Avantor), penicillin (1 unit/ml), streptomycin (1 μg/ml), glutamine (2 mM) (Thermo Fisher), sodium pyruvate (1 mM) (Thermo Fisher), and 1X Minimal Essential Media Non-Essential Amino Acids (Thermo Fisher). Before use, we passed the media through a 0.22 um vacuum filter (Falcon). Jurkat cells are maintained in RPMI 1640 with Glutamax and HEPES (Gibco), supplemented with 100X diluted pen/strep (Thermo Fisher) with 10% FBS and sodium pyruvate (100X). THP-1 cells (a gift from Mikhail Shapiro’s lab at Caltech) and GSDMD-knockout THP-1 cells (a gift from Hao Wu’s lab at Harvard) were maintained in RPMI 1640 with Glutamax and HEPES (Gibco), supplemented with 100X diluted pen/strep (Thermo Fisher) and 10% FBS. For routine passage or seeding, we lifted HEK cells from plates using 0.05% Trypsin-EDTA (Thermo Fisher). Jurkat and THP-1 cells were resuspended by vigorous pipetting and did not require trypsinization. HEK cells were maintained at a confluence between 10% and 90% by estimation. Jurkat and THP-1 cells were maintained at a density between 200,000 cells/ml and 2 million cells/ml. All cells were used before they reached 30 passages and then discarded. The volume of media was 100 ul to 200 ul per well on a 96-well plate, 500 ul to 1 ml per well on a 24-well plate, 1 ml - 2 ml per well on a 12-well plate, 2–3 ml per well on a 6-well plate, and 10 ml - 12 ml on a 10-cm dish. Regarding sex, HEK cells are female, Jurkat cells are male, and THP-1 cells are male. We verified the cells to be free of mycoplasma by polymerase chain reaction (PCR) using the MycoStrip kit (InvivoGen).

### METHOD DETAILS

#### Study design

The objective of this study was to engineer protein-level synthetic synpoptosis circuits that enable programmable control of cell death in mammalian cells. We designed and performed molecular biology and cell biology experiments to investigate the capabilities of synpoptosis circuits. Results shown represent three independent replicates, as indicated in figure legends. This study was not blinded.

#### Plasmid construction

We used the NEBuilder HiFi DNA Assembly Master Mix (New England BioLabs) to assemble gene inserts, synthesized (Integrated DNA Technologies) or amplified from previous plasmids33 in the lab, into restriction digested in-house cloning vectors. We designed the insert sequences for expression in human cells using a codon optimization tool (Integrated DNA Technologies). To generate mutant plasmids, we performed Gibson assembly of synthesized mutant genes or used the Q5 Site-Directed Mutagenesis Kit (New England BioLabs). For transfection, we purified the plasmids manually using the QIAprep Spin Miniprep Kit (Qiagen) or by the Qiacube machine (Qiagen). To verify their sequences before use, we sent the plasmids to Primordium or Laragen.

#### mRNA production

Genes of interest were cloned into a DNA backbone containing a T7 promoter followed by an AG dinucleotide. The DNA templates were linearized and a 3’ 120-base pair (bp) polyA tract was added using PCR. The PCR product was run on an agarose gel and cleaned using the Zymoclean Gel DNA Recovery Kit (Zymo). In vitro transcribed mRNA was then synthesized using the HiScribe T7 High Yield RNA Synthesis kit (NEB). Reactions consisted of a 10X Reaction Buffer, 5 mM each of ATP, GTP, CTP, and N1-Methyl-Pseudouridine-5’-Triphosphate (TriLink), 4 mM CleanCap AG (TriLink), 500 ng of linearized DNA template, 2 μL of T7 RNA polymerase mix, and water for a final volume of 20 μL. Reactions were incubated for 2 hours at 37 °C. After incubation, 5 μL of DNAse I buffer, 1 μL of DNAse I and 25 μL of water were added to the reactions. Reactions were then incubated for 15 minutes at 37 °C. Finally, mRNA was purified using the RNA Clean and Concentrator-5 kit (Zymo).

#### Transient transfection

On the day before transfection, HEK cells were seeded at a density of 0.3–0.6 million cells per well on a 12-well plate, 0.1–0.3 million cells per well on a 24-well plate, or 15,000–50,000 cells per well on a 96-well plate. The cells were allowed to attach to the plate overnight, for at least 8 hours. On the day of transfection, we treated the cells with purified DNA plasmids encoding circuit components. In addition to plasmids encoding circuit components, we often included a plasmid encoding a fluorescent co-transfection marker, at roughly 50 ng DNA per well on a 24-well plate. Given the different number of molecular components in each synthetic circuit and their various relative ratios, the total amount of DNA ranged from 50 to 1 μg per well on a 24-well plate, and was scaled by 0.5 or 4 when other 12-well or 96-well plates were used, respectively. On the same plate, we often equalized the total amount of DNA across all transfected wells, using a filler plasmid encoding the neomycin resistance gene. To transfect cells with plasmid DNA, we used three types of transfection reagents – Lipofectamine 2000 (Thermo Fisher), Lipofectamine 3000 (Thermo Fisher), and FuGENE HD (Promega), according to manufacturers’ instructions. Briefly, for a typical transfection experiment on a 24-well plate, we mixed plasmids and the transfection reagent, around 200 ng of DNA per 1 μl of Lipofectamine 2000, in 25 μl of Opti-MEM reduced serum medium (Thermo Fisher), incubated the mixture for 5–10 min at room temperature, and then added another 25 μl of Opti-MEM before transferring the mixture dropwise to culture wells. We also performed some transfection experiments using the Lipofectamine 3000 kit (Thermo Fisher), at a ratio of 2,000 ng of DNA, 2 ul of 4 ul of P3000, and 3 μl of Lipofectamine 3000. We similarly diluted the mixture in Opti-MEM before adding it to cells. For transfection with FuGENE, we used a ratio of 4 μl FuGENE:1 μg of DNA, and incubated the DNA-FuGENE mixture at room temperature for 5 min before transfection. To enable protein expression from plasmids with a doxycycline-inducible promoter, we added doxycycline to the cell culture at a final concentration of 100 ng/mL. In experiments involving Q-VD-OPh, the compound was added to the cell culture at a final concentration of 30 μM, immediately after the cells received the transfection mixture. To transfect cells with in vitro transcribed mRNA, we used Lipofectamine MessengerMax (Thermo Fisher). Briefly, for transfection of one well in a 96-well plate, 0.1 μL of Lipofectamine MessengerMAX was diluted in 1.25 μL Opti-MEM and incubated for 10 minutes at room temperature. mRNA was then added to Opti-MEM to the appropriate conditions at the following amounts: 12.5 ng of fluorescent co-transfection marker, 56.25 ng of protease, and 56.25 ng of engineered executioner. Diluted mRNA was then added to diluted MessengerMAX reagent and incubated for 5 minutes at room temperature before addition to Jurkat or THP-1 cells in RPMI media.

#### Cell line engineering

For experiments that required stable transgene expression, we engineered stable cell lines using lentiviruses followed by antibiotic selection, or using the PiggyBac transposase system. To generate lentiviruses, we plated HEK293 cells (maintained in geneticin at 500 μg/mL) at a density of 0.6 million cells per well on a 12-well plate, or to around 90% confluence by estimation in a T25 flask. The next day, we co-transfected the cells using Lipofectamine 2000 with three plasmids, pLVX-M-PURO (Addgene plasmid #125839) containing the gene of interest, the TEVP-activatable Citrine reporter for instance, psPAX2 (Addgene plasmid #12260), and pCMV-VSV-G (Addgene plasmid #8454). We kept the mass ratio of the three plasmids at 9:9:2 (450 ng:450 ng:100 ng). In some experiments involving cells stably expressing two or more transgenes, the lentiviruses were generated using multiple pLVX-M-PURO plasmids carrying different genes of interest. An alternative ratio of pLVX-M-PURO:psPAX2:pCMV-VSV-G was 2:2:1 (2.4 μg:2.4 μg:1.2 μg), which we used to generate lentiviruses for stable integration of IL-1β and IL-18 genes. 48 hours post transfection, we collected supernatants containing the lentiviruses and used them to transduce HEK cells, 0.3 million per well, on a 24-well plate. We then waited 48 hours to allow integration and transgene expression in treated cells. Afterwards, we transferred the transduced cells to a 6-well plate and added puromycin (1 μg/ml, Sigma-Aldrich) to select polyclonal cells with successfully integrated transgenes over a period of two weeks, during which we refreshed the media every two days. To generate control cells, we used empty lentiviruses. The RasGC line, or HEK293 cells stably expressing human H-Ras G12V mutant along with a Cerulean fluorescence marker, was generated using the PiggyBac method. The mutant Ras and Cerulean marker were cloned into a PiggyBac DNA vector connected by a 2A peptide. Expression of the mutant Ras and Cerulean marker was driven by a dox-inducible CMV promoter. Briefly, 50 ng of the PiggyBac transposase and 200 ng of the cargo-carrying DNA vector were co-transfected into HEK293 cells seeded in a 24-well plate. One week later, the cells were sorted based on Cerulean fluorescence to obtain the final RasGC line.

#### Intercellular transmission

As a model VLP that demonstrates intercellular transmission of synpoptosis circuits, we used integrase-deficient lentiviruses. We built the VLP packaging plasmid by introducing the inactivating D64V mutation into the integrase gene within the standard lentiviral vector psPAX2 (hereafter, modified psPAX2, or M-psPAX2). For experiments that involved circuit senders and receivers, we seeded sender HEK cells on a 12-well plate at a density of 0.6 million cells per well (day 1). On the following day (day 2), using Lipofectamine 3000 (Thermo Fisher), we transfected the sender cells with 450 ng of pLVX-M-PURO plasmid containing the cargo, 450 ng of M-psPAX2, and 100 ng of pCMV-VSV-G. To generate VLPs encoding two cargoes, we performed similar transfections using two cargo-containing plasmids, each at 225 ng. At 8 hours post transfection, we removed the media containing residual Lipofectamine particles, washed the cells gently with fresh media, and replaced the media. One day after transfection of sender cells (day 3), we typically seeded receiver HEK cells on a 24-well plate at a density of around 0.3 million cells per well or on a 96-well plate at around 75,000 cells per well. We also tried other receiver densities, in which case we needed to dilute the cells if they approached confluency during the course of the experiment. Two days after transfection of sender cells (day 4), we collected the sender supernatant, centrifuged it at 3,000 g for 5 min to remove cell debris, and passed it through a cellulose acetate filter with 0.45 μm pore size (VWR). We transferred the VLP-containing conditioned media, without concentrating them, to receiver cells. One day after media transfer (day 5), we refreshed the media. Finally, two days after media transfer (day 6), we collected receiver cells for further analysis. In co-culture experiments, sender HEK cells were pre-stained with Vybrant DiD (Invitrogen) by incubation at 37 °C for 15 min and washed three times to remove the unbound stain, according to manufacturer’s instructions. Then, the senders were transiently transfected with DNA encoding the silencer, VLP-packaging components, and the active executioner cargo (GSDMA N-Z, or empty cargo as control). 8 hours post transfection, the supernatant was discarded and the transfected senders were washed twice with DPBS. Then, the senders were lifted by trypsinization and mixed with receivers, which were not stained, at a roughly 1:1 cell number ratio in a 24-well plate. After about 40 hours, we collected the co-cultured cells, stained them with Sytox, and analyzed cell death by flow cytometry.

#### Cell staining and flow cytometry

For experiments involving DNA transfection, we collected the cells from culture wells between 16 and 24 hours post transfection, a selected time window during which substantial cell death occurs while apoptotic cells remain largely unlysed, allowing Sytox to reliably distinguish between apoptotic and pyroptotic cells. To collect all cells, we first transferred the supernatant containing floating cells to a 1.5-ml Eppendorf tube or a 15-ml Falcon tube. Then, we gently washed the adherent cells with Dulbecco’s phosphate-buffered saline (DPBS, Gibco) and trypsinized them using 0.05% Trypsin-EDTA (ThermoFisher) for 5 min at room temperature. Afterwards, we pooled the trypsinized cells with the supernatant and pelleted the cells in a tabletop centrifuge (Eppendorf 5424 or 5804R) at 200 g for 5 min. We then carefully removed the supernatant and resuspended the pellet in 1.5 ml of the Hank’s Balanced Salt Solution (HBSS, Gibco) containing 2.5 mg/ml bovine serum albumin (BSA) and 2.5 mM calcium chloride. For Sytox staining, we used one drop of the SYTOX Green Ready Flow reagent (R37168, Invitrogen). For Annexin staining, we used one drop of Annexin Ready Flow reagents, conjugated with either Pacific Blue (R37177) or Alexa Fluor 488 (R37174). For staining with both dyes, we added one drop of each. For TO-PRO-3 staining, an indicator of apoptosis or membrane damage during pyroptosis, we used TO-PRO-3 (Invitrogen T3605) at a final concentration of 1 μM. Afterwards, we incubated the cells on ice in the dark for 15 min. For experiments involving lentiviruses or VLPs, we collected cells for staining around 2 days post treatment with lentiviruses or VLPs. For experiments involving mRNA transfection, we collected cells for staining between 8 and 12 hours post transfection. To analyze cells by flow cytometry, we filtered the collected and stained cells through a 40-μm cell strainer (Falcon) and then transferred them to a U-bottom 96-well plate on top of a cold block. The volume in each well was 150 to 250 μl. We then subjected the cells to analysis by the CytoFLEX S flow cytometer (Beckman Coulter), with forward and side scatters set at 185 and 115 (other similar settings were also used). Mainly four channels recorded cellular fluorescence of interest in the study, the FITC channel (for Citrine, Sytox Green, and Annexin-Alexa Fluor 488; excitation 488 nm, emission 525/40 nm; gain set at 1), the ECD channel (for Cherry; excitation 561 nm, emission 610/20 nm; gain set at around 50–100), the PB450 channel (for Annexin-Pacific Blue; excitation 405 nm, emission 450/45 nm; gain set at around 50–100), and the APC channel (for Vybrant DiD; excitation 638 nm, emission 660/20; gain set at around 100). For experiments involving multiple colors, we collected single-color controls to set the compensation matrix, using built-in functions in the FlowJo software (version 10.4, BD Biosciences). Gray windows or horizontal lines that mark y-axis ranges in the figures were established by negative and positive transient transfection controls, established by mock transfection (lipofectamine only, transfection of Cherry only, or transfection of an inactive circuit containing a mutant executioner, such as caspase-3-C163A or GSDMA-E14K-L184D mutant, and the TEVP-C151A mutant) and transfection of a constitutively active killer (protease-activated circuit or an active executioner), respectively. In transfection experiments involving incoherent feedforward loop (IFFL)-regulated GFP expression, cells were left unstained, and the gray window indicates negative and positive controls of the GFP signal, set by mock transfection and transfection of GFP without IFFL regulation, respectively. In experiments involving a fluorescent co-transfection marker, such as Cherry, we often gated on the co-transfection marker to analyze the effects of synpoptosis circuits on transfected cells. Some cells were Annexin-positive and Cherry-negative. These cells were of several possible origins and excluded from analysis.

#### Cell imaging

We examined live and dead cells using the EVOS FL Auto cell imaging system (Life Technologies) approximately 16–24 hours post DNA transient transfection. The three EVOS light cubes correspond to cyan fluorescence protein (excitation 445/45 nm, emission 510/42 nm), yellow fluorescent protein (excitation 500/24 nm, emission 542/27 nm), and red fluorescent protein (excitation 531/40 nm, emission 593/40 nm). For magnification, we typically used the EVOS AMG 10x or 20x objective. We usually set the exposure at around 200 milliseconds and the gain at around 10 decibels. Images were captured as tiff files for quantification purposes. Images within a figure panel were presented using the same brightness and color scales.

#### Cell viability and cytotoxicity experiments

For ATP-based viability experiments, 15,000 HEK293 cells were seeded to each well of an opaque 96-well plate and incubated overnight under standard culture conditions. After 24 hours, the cells were transfected with 100 ng in vitro transcribed mRNA, previously prepared using the Lipofectamine MessengerMax transfection kit as per the manufacturer’s protocol (0.3 μL Lipofectamine per 100 ng mRNA, in 12.5 μL total volume of mRNA-Lipofectamine transfection mix). Cells were incubated for another 8 hours before their total ATP levels were measured using the CellTiter-Glo 2.0 cell viability assay (Promega). Both cells and CellTiter-Glo 2.0 reagent were equilibrated to room temperature before use. 100 μL of the assay reagent was added to 100 μL of sample medium per well. After mixing by orbital shaking (1 mm shaking radius, 300 cycles per minute, 2 min), the plate was incubated for 10 min at RT and luminescence was subsequently recorded using a GloMax Discover microplate reader (Promega) without a filter and with a 0.7-second integration time. ATP-based cell viability was calculated by dividing the luminescence of the compound treated by the luminescence of untreated sample wells. For LDH release experiments, the same number of cells were seeded into a standard 96-well plate, and after 24 hours, transfected with the same amounts of mRNA using the same transfection procedures. Wells used to determine the levels of spontaneously released LDH were transfected with Opti-MEM only. At 8 hours post transfection, we used the Invitrogen’s CyQUANT LDH cytotoxicity assay kit (Invitrogen) to measure LDH release per well. The reaction reagent was prepared by adding ddH2O (11.4 mL) and assay buffer stock solution (0.6 mL) to the substrate mix. To determine the maximum LDH activity, 10X lysis buffer (10 μL) was added to corresponding wells serving as maximum LDH activity controls. After 45 min incubation under standard culture conditions, 50 μL of supernatant sample medium per well was transferred to a black-walled clear bottom 96-well plate. After adding 50 μL reaction mixture reagent per well, the plate was mixed and incubated at room temperature for 30 min. Formazan production, proportional to LDH amounts, was stopped by supplementing 50 μL stop solution per well. Absorbance at 490 nm and 680 nm was then measured using a BioTek Cytation 5 cell imaging multimodal reader (Agilent). LDH release was calculated by subtracting the spontaneous from circuit-treated LDH activity and subsequent division by the difference between maximum and spontaneous LDH activity. For ATP release experiments, the same number of cells were seeded into an opaque 96-well plate and transfected the same way with mRNA after 24 hours. ATP release was measured using the RealTime-Glo Extracellular ATP assay (Promega). Briefly, 4x RealTime-Glo Extracellular ATP assay reagent was prepared by adding supplemented, pre-warmed DMEM culture medium (Gibco) to RealTime-Glo Extracellular ATP assay substrate. 50 μL of the assay reagent was added to 150 μL sample medium per well, and the plate was mixed by orbital shaking (2 mm shaking radius, 300 cycles per minute, 45 s). Immediately after adding the assay reagent, luminescence was recorded every hour over 20 hours using a GloMax Discover microplate reader (Promega) without a filter. The integration time was 0.7 seconds, and the incubation temperature was 37 °C.

#### Cytokine measurements

Concentrations of IL-1β and IL-18 in the cell culture supernatant were measured using enzyme-linked immunosorbent assays (ELISA). HEK293 cells stably expressing IL-1β and IL-18 were seeded into 24-well plates at 150,000 cells per well and then transiently transfected with lipofectamine only (mock transfection control) or 300 ng of DNA-encoded TEVP-activated apoptosis or pyroptosis circuits. Between 16 and 24 hours post-transfection, supernatants were collected into 1.7-ml tubes and spun at 15,000 g for 5 min using a tabletop centrifuge (Eppendorf). Clarified supernatants were transferred to clean 1.5-ml tubes, and then diluted 100- to 1000-fold before ELISA using SimpleStep IL-1β and IL-18 ELISA kits (Abcam). Briefly, 50 μl of diluted samples and 50 μl of the antibody cocktail solution, which contained both capture and detection antibodies, were added to each well of the microplate strips. The strips were incubated for around 1 hour at room temperature on a shaker. Each well was washed with 350 μl of wash buffer three times before 100 μl of development solution containing TMB (tetramethylbenzidine), a substrate for horseradish peroxidase (HRP), was added. The strips were covered in foil and shaken for 10 min at room temperature. 100 μl of stop solution was added to each well to quench the reaction. Absorbance at 450 nm was recorded using a Cytation 3 plate reader (BioTek). Diluted IL-1β and IL-18 concentrations were calculated based on standard curves established using lyophilized purified proteins dissolved to known concentrations. Undiluted concentrations were then obtained by multiplying the diluted concentrations by the corresponding dilution factor.

#### Protein structure analysis

To evaluate molecular constraints on the design of synpoptosis circuit components, we analyzed several structures from the protein data bank (PDB) database, which can be located by the following accession numbers. These structures include cryo-electron microscopy (cryo-EM) structures of GSDM pores (PDB 6CB8, 6VFE, 8GTN, 8ET2, and 8SL0),^54–58^ as well as X-ray crystal structures of GSDMA (GSDMA3 variant) (PDB 5B5R),^100^ GSDMD (PDB 6N9O),^101^ bGSDM (PDB 7N51),^102^ caspase-3 (PDB 1I3O),^103^ TEVP (PDB 1Q31),^104^ and TVMVP (PDB 3MMG).^105^ To examine molecules whose structures are not available, such as engineered GSDMA containing a TEVP cleavage site or leucine zippers, we generated models using AlphaFold2 (ColabFold v1.5.2).^106^ For molecular visualization, we used PyMOL software (2.5.2) (Schrodinger).^107^

### QUANTIFICATION AND STATISTICAL ANALYSIS

Sample sizes and analytical measures are indicated in the figure legends and method details. To generate data visualizations, we used ImageJ,^108^ Excel (Microsoft), the built-in layout editor in FlowJo (version 10.4, BD Biosciences), GraphPad Prism (version 9, Dotmatics), and Matplotlib (Python).^109^ Sigmoidal curve was fitted using the four parameter logistic (4PL) nonlinear regression method in GraphPad Prism. For plots that required coding, we used the Jupyter Notebook (jupyter.org) with the assistance of ChatGPT (version 4, OpenAI). To reduce visual overload, each flow cytometry scatter plot shows 5,000 randomly sampled data points, and each flow cytometry histogram shows the fluorescence distribution of 5,000 randomly sampled data points divided into 50 bins on the x-scale. In plots that quantify fractional killing, the upper and lower limits of signal quantification were defined by positive and negative controls, respectively, as indicated in method details. In transfection experiments involving a fluorescent co-transfection marker, we often gated on the co-transfection marker by flow cytometry to identify transfected cells before quantifying killing fractions. This approach allowed us to attribute cell death to synpoptosis circuits.

## Supplementary Material

1 **Figure S1. Synpoptosis circuits orthogonally control cell death, related to** Figure 1
**(A)** HEK cells express negligible levels of endogenous GSDMs. Expression data were obtained from the Human Protein Atlas (proteinatlas.org).
**(B)** We performed the majority of experiments in the paper using the transient transfection method, as shown in the schematic with mock data. We co-transfected the cells with plasmid DNA encoding the synthetic circuits and a fluorescent protein marker. After 16–24 hours post-transfection, we collected both floating and attached cells for staining and flow cytometry.
**(C)** There are several possible sources of cells that stain low for the fluorescent co-transfection marker (Cherry) and high for the death dye (Annexin). First, the transfection reagent itself causes some toxicity; second, the cell collection procedure involving pelleting and resuspending kills some cells; and third, when the circuits kill the cells, they also reduce the level of the fluorescent protein in the cells, making some transfected cells appear Cherry-low. Given the uncertain origins, we focused on Cherry-high cells by gating, which allowed us to definitively attribute the observed cell death to the transfected synthetic circuits. Data represent three independent experiments.
**(D)** Three viral proteases, TEVP, TVMVP, and HCVP, orthogonally activated their cognate engineered GSDMs containing the cleavage sites (tev, tvmv, and hcv, respectively). Data represent three independent experiments. Colors indicate means.
**Figure S2. Synpoptosis circuits show typical features of natural death programs, related to** Figure 2
**(A)** In transient DNA transfection experiments, engineered auto-inhibited caspase-3 or GSDMA induced modest cell death when they were highly expressed, without the activating TEVP. Within each bin on the x-axis, dots represent biological replicates (distinct culture wells).
**(B)** Titrating the DNA amounts of the circuit plasmids enabled dose-dependent control over the fraction of cell killing. Throughout the figure, the gray window indicates the y-ranges established by positive and negative transient DNA transfection controls; dots represent biological replicates (distinct culture wells).
**(C)** At a constant amount of the plasmid DNA, we could tune the penetrance of synpoptosis circuits by modulating protein expression at the mRNA level, using a synthetic miRNA-based incoherent feedforward loop (IFFL). The strength of mRNA inhibition by the miRNA is tunable by adjusting base complementarity between mRNA and miRNA. In the GFP panel, dots mark median fluorescence values.
**(D)** Cells killed by synpoptosis circuits displayed canonical morphological characteristics of apoptosis and pyroptosis. Images are representative of three independent experiments and on the same brightness and color scales. Scale bars: 200 μm (left) and 20 μm (right).
**Figure S3. Synpoptosis circuits modulate cell death, related to** Figure 3
**(A)** GSDME mutants displayed various pyroptotic activity compared to wildtype GSDME N-domain (N), shown by transient DNA transfection and flow cytometry. The last mutant was defective and later referred to as GSDME N(mut). GSDME N(mut) inhibited wildtype GSDME N-induced pyroptosis. Ratio of mutant to wildtype GSDME (mass of DNA plasmids) from left to right: 1-to-1 (low), 2-to-1 (medium), and 4-to-1 (high). The gray window indicates the fractions established by negative and positive transient DNA transfection controls. Throughout the figure, dots represent biological replicates (distinct culture wells).
**(B)** Time-course flow cytometry experiments suggested that GSDME N(mut) promoted apoptosis to various degrees, shown by different Sytox levels and similar Annexin levels, in cells expressing wildtype GSDME. The gray window indicates that Sytox remains low in apoptotic cells between 16 and 24 hours post DNA transfection.
**(C)** Using GSDMD-KO THP-1 cells, we demonstrated that the mRNA-encoded synpoptosis circuits trigger the expected mode of cell death, independently of endogenous death circuitry. Horizontal lines indicate the fractions established by negative and positive transient mRNA transfection controls.
**Figure S4. Synpoptosis circuits enable complex functions, related to** Figure 4
**(A)** We built more gates that synthetically control apoptosis based on different protease input combinations, shown by transient DNA transfection and flow cytometry experiments. Throughout the figure, the gray window indicates the fractions established by negative and positive transient DNA transfection controls; dots represent biological replicates (distinct culture wells).
**(B)** The corresponding logic gates for synthetic control of pyroptosis were constructed similarly.
**Figure S5. Synpoptosis circuits exhibit target cell selectivity, related to** Figure 5
**(A)** Proximity-reconstituted TEVP in RasGC cells should protect a degron-tagged Citrine reporter (Citrine-tev-Deg) from degradation, enhancing Citrine fluorescence.
**(B)** The synthetic Ras sensor enabled selective targeting of RasGC cells, shown by RasGC-specific enhancement of Citrine fluorescence, measured by flow cytometry after transient DNA transfection. Light and dark gray colors indicate WT and RasGC cells, respectively. The Citrine-tev-Deg reporter was stably integrated into RasGC and WT cells by lentiviral transduction. Data represent three independent experiments.
**(C)** The combination of nTEVP-RBD and cTEVP-RBD enhanced Citrine fluorescence in RasGC cells, shown on both linear and log scales. Horizontal lines indicate the median fluorescence values established by negative and positive transient DNA transfection controls, established separately for each cell line; bars indicate the means of median fluorescence values; dots represent biological replicates (distinct culture wells).
**Figure S6. Synpoptosis circuits allow cell-cell transmission, related to** Figure 6
**(A)** VLPs expressing a circuit component could complement the missing component in the cell, shown by flow cytometry after supernatant transfer. The gray window indicates the fractions established by negative and positive transient DNA transfection controls. Throughout the figure, dots represent biological replicates (distinct culture wells), and bars indicate means.
**(B)** Inhibition of GSDMA N by GSDMA C requires a zipper (Z) on each of the two molecules, shown by transient DNA transfection and flow cytometry experiments. The gray window indicates the fractions established by negative and positive transient DNA transfection controls.
**(C)** Senders expressing GSDMA Z-C (the silencer) after transient DNA transfection were protected from pyroptosis when treated with supernatants containing VLPs that express GSDMA N-Z (the engineered active executioner). The gray window indicates the fractions established by wildtype senders treated with empty VLPs and GSDMA N-Z VLPs, respectively.
**(D)** GSDMA Z-C (the silencer) suppressed cytotoxicity caused by GSDMA N-Z (the engineered active executioner). Images are representative of three independent experiments and on the same brightness and color scales. Scale bar: 200 μm.
**(E)** The single-sender system functions in a co-culture context. Sender cells were transiently transfected with DNA to express the silencer and package GSDMA N-Z VLPs or empty VLPs as a control. The senders, but not receivers, were stained with Vybrant DiD. To show that Vybrant DiD persists in dead cells, we transfected HEK cells pre-stained by Vybrant DiD with plasmid DNA encoding GSDMA N-Z, followed by Sytox staining and flow cytometry.

## Figures and Tables

**Table:** KEY RESOURCES TABLE

REAGENT or RESOURCE	SOURCE	IDENTIFIER
Bacterial and virus strains		
Stable Competent *E. coli* (High Efficiency)	New England Biolabs	Cat# C3040H
10-beta Competent *E. coli* (High Efficiency)	New England Biolabs	Cat# C3019H
Chemicals, peptides, and recombinant proteins		
Dulbecco’s Modified Eagle Medium	ThermoFisher	Cat# 11960-069
Roswell Park Memorial Institute (RPMI) 1640 Medium with GlutaMAX supplement, HEPES	Gibco	Cat# 72400-047
Fetal bovine serum (FBS)	Avantor	Cat# 97068-085
Penicillin-Streptomycin-Glutamine	ThermoFisher	Cat# 10378-016
Sodium pyruvate	ThermoFisher	Cat# 11360070
Minimal Essential Medium Non-Essential Amino Acids	ThermoFisher	Cat# 11140050
Trypsin-EDTA (0.05%)	ThermoFisher	Cat# 25300054
Trypsin-EDTA (0.25%)	ThermoFisher	Cat# 25200056
Dulbecco’s Phosphate Buffered Saline (DPBS) containing calcium and magnesium	ThermoFisher	Cat# 14040117
Hank’s Balance Salt Solution (HBSS)	Gibco	Cat# 14025134
Bovine serum albumin (BSA)	Sigma Aldrich	Cat# A4503
Calcium chloride	J.T. Baker	Cat# 1311-01
Opti-MEM I Reduced Serum Medium	ThermoFisher	Cat# 31985-070
Carbenicillin, disodium salt	Invitrogen	Cat# 10177-012
Kanamycin Monosulfate	Goldbio	Cat# K-120-100
Trypsin	Promega	Cat# VA9000
Q-VD-OPh	Cayman Chemical	Cat# 15260
Poly-D-Lysine	ThermoFisher	Cat# A3890401
Puromycin Dihydrochloride	ThermoFisher	Cat# A1113803
Hygromycin B Gold	InvivoGen	Cat# ANT-HG-1
Doxycycline Hydrochloride	Sigma Aldrich	Cat# D3072
Lipofectamine 2000	ThermoFisher	Cat# 11668019
Lipofectamine 3000	ThermoFisher	Cat# L3000008
FuGENE HD	Promega	Cat# E2311
Lipofectamine MessengerMAX	ThermoFisher	Cat# LMRNA001
Triton X-100	Fisher Scientific	Cat# AC327371000
Sytox Green Ready Flow	Invitrogen	Cat# R37168
Annexin V Pacific Blue Ready Flow	Invitrogen	Cat# R37177
Annexin V Alexa Fluor 488 Ready Flow	Invitrogen	Cat# R37174
TO-PRO-3 Iodide	Invitrogen	Cat# T3605
Trypan Blue Stain, 0.4%	Invitrogen	Cat# T10282
Vybrant DiD	Invitrogen	Cat# V22887
LB Broth with agar (Lennox)	Sigma Aldrich	Cat# L2897-250G
Terrific Broth Powder	Research Products International	Cat# T15000
50x TAE buffer	Fisher Scientific	Cat# 50-146-818
Agarose I (Molecular Biology Grade)	Fisher Scientific	Cat# 17852
Blue gel loading dye	New England Biolabs	Cat# B7021S
SyBrSafe	Fisher Scientific	Cat# S33102
1-kb DNA ladder	New England Biolabs	Cat# N3232L
T4 ligase	New England Biolabs	Cat# M0202M
DMSO	Millipore Sigma	Cat# D8418
Critical commercial assays		
Gibson Assembly Master Mix	New England Biolabs	Cat# E2611L
QIAprep Spin Miniprep Kit	Qiagen	Cat# 27104
Zymoclean Gel DNA Recovery Kit	Zymo Research	Cat# D4007
HiScribe T7 High Yield RNA Synthesis Kit	New England Biolabs	Cat# E2040S
RNA Clean and Concentrator-5 Kit	Zymo Research	Cat# R1013
QIAquick Gel Extraction Kit	Qiagen	Cat# 28706
CellTiter-Glo Luminescent Cell Viability Assay	Promega	Cat# G7570
CyQUANT LDH Cytotoxicity Assay Kit	Invitrogen	Cat# C20201
RealTime-Glo Extracellular ATP assay Kit	Promega	Cat# GA5011
SimpleStep IL-1 beta ELISA kit	Abcam	Cat# ab197742
SimpleStep IL-18 ELISA kit	Abcam	Cat# ab216165
MycoStrip	InvivoGen	Cat# rep-mysnc-50
Experimental models: Cell lines		
HEK293 cell line	ATCC	Cat# CRL-1573
HEK293T cell line	ATCC	Cat# CRL-3216
HEK293FT cell line	ThermoFisher	Cat# R70007
T-REx-293 cell line	ThermoFisher	Cat# R71007
HEK293T cells with stable expression of TEVP and Cherry	This paper	N/A
HEK293T cells with stable expression of TVMVP and Citrine	This paper	N/A
HEK293T cells with stable expression of TEVP, TVMVP, Cherry, and Citrine	This paper	N/A
HEK293T cells with stable expression of IL-1beta and IL-18	This paper	N/A
HEK293 cells with stable expression of H-RAS G12V mutant and Cerulean (RasGC cells)	This paper	N/A
RasGC line with stable expression of TEVP-activatable Citrine reporter	This paper	N/A
HEK293 cells with stable expression of TEVP-activatable Citrine reporter	This paper	N/A
Jurkat cell line	ATCC	Cat# TIB-152
THP-1 cell line	A gift from Mikhail Shapiro’s lab (Caltech)	N/A
GSDMD KO THP-1 cells	A gift from Hao Wu’s lab (Harvard)	N/A
Recombinant DNA		
CMV-TO-TEVP	Gao et al.^33^	As described^33^
CMV-TO-TVMVP	Gao et al.^33^	As described^33^
CMV-TO-scHCVP	Gao et al.^33^	As described^33^
pSCD-TEVP	This paper	N/A
pSCD-TVMVP	This paper	N/A
pSCD-NS4HCVP	This paper	N/A
pSCD-H2B-Cherry	This paper	N/A
pSCD-ZeoR	This paper	N/A
pSCD-Casp3-L-tev-S	This paper	N/A
pSCD-Casp3-Z-L	This paper	N/A
pSCD-Casp3-S-Z-tev-Deg	This paper	N/A
pSCD-Casp3-tevD-Z-L	This paper	N/A
pSCD-Casp3-S-Z	This paper	N/A
pSCD-GSDMA-N	This paper	N/A
pSCD-GSDMA-C	This paper	N/A
pSCD-GSDMA-N-tev-C	This paper	N/A
pSCD-GSDMA-MBP-tev-N	This paper	N/A
pSCD-GSDMA-N-tev-Deg	This paper	N/A
pSCD-GSDMA-N-tev	This paper	N/A
pSCD-GSDMA-tevD-N	This paper	N/A
pSCD-GSDMA-N-tvmv-C	This paper	N/A
pSCD-GSDMA-N-hcv-C	This paper	N/A
pSCD-GSDMD-N-tev-C	This paper	N/A
pSCD-GSDMD-N-tvmv-C	This paper	N/A
pSCD-GSDMD-N-hcv-C	This paper	N/A
pSCD-GSDME-N-tev-C	This paper	N/A
pSCD-GSDME-N-tvmv-C	This paper	N/A
pSCD-GSDME-N-hcv-C	This paper	N/A
pSCD-Casp3-L-tev-S-2A-TEVP	This paper	N/A
pSCD-GSDMA-N-tev-C-2A-TEVP	This paper	N/A
pSCD-IVT-H2B-Cherry	This paper	N/A
pSCD-IVT-TEVP	This paper	N/A
pSCD-IVT-Casp3-L-tev-S	This paper	N/A
pSCD-IVT-GSDMA-N-tev-C	This paper	N/A
pSCD-IFFL-GFP-noreg	This paper	N/A
pSCD-IFFL-GFP-noreg-18×4	This paper	N/A
pSCD-IFFL-GFP-noreg-19×4	This paper	N/A
pSCD-IFFL-Casp3-L-tev-S-2A-TEVP-noreg	This paper	N/A
pSCD-IFFL-Casp3mut-2A-TEVPmut-noreg	This paper	N/A
pSCD-IFFL-Casp3-L-tev-S-2A-TEVP-18×4	This paper	N/A
pSCD-IFFL-Casp3-L-tev-S-2A-TEVP-19×4	This paper	N/A
pSCD-IFFL-GSDMA-N-tev-C-2A-TEVP-noreg	This paper	N/A
pSCD-IFFL-GSDMAmut-2A-TEVPmut-noreg	This paper	N/A
pSCD-IFFL-GSDMA-N-tev-C-2A-TEVP-18×4	This paper	N/A
pSCD-IFFL-GSDMA-N-tev-C-2A-TEVP-19×4	This paper	N/A
pSCD-GSDME-FLwt	This paper	N/A
pSCD-GSDME-Nwt	This paper	N/A
pSCD-GSDME-N-V99N	This paper	N/A
pSCD-GSDME-N-L101N	This paper	N/A
pSCD-GSDME-N-L103N	This paper	N/A
pSCD-GSDME-N-V191E	This paper	N/A
pSCD-GSDME-N-V193E	This paper	N/A
pSCD-GSDME-N-A195E	This paper	N/A
pSCD-GSDME-N-G199E	This paper	N/A
pSCD-GSDME-N-I217N	This paper	N/A
pSCD-Casp3-L-tev-S-tvmv-Deg	This paper	N/A
pSCD-Casp3-L-tev-tvmv-S	This paper	N/A
pSCD-Casp3-tvmvD-Z-L	This paper	N/A
pSCD-GSDMA-MBP-tvmv-N-tev-C	This paper	N/A
pSCD-GSDMA-N-tev-tvmv-C	This paper	N/A
pSCD-GSDMA-tvmvD-N-tev-Deg	This paper	N/A
pSPAX2	Addgene	Cat# 12260
pMD2.G	Addgene	Cat# 12259
pCMV-VSV-G	Addgene	Cat# 8454
pSCD-LV-TEVP	This paper	N/A
pSCD-LV-TVMVP	This paper	N/A
pSCD-LV-Cherry	This paper	N/A
pSCD-LV-Citrine	This paper	N/A
pSCD-LV-TEVP-2A-Cherry	This paper	N/A
pSCD-LV-TVMVP-2A-Citrine	This paper	N/A
pSCD-Casp3-tevD-L-tvmv-S	This paper	N/A
pSCD-Casp3-tvmvD-L-tev-S	This paper	N/A
pSCD-Casp3-tevD-tvmv-Z-L	This paper	N/A
pSCD-GSDMA-tevD-N-tvmv-C	This paper	N/A
pSCD-GSDMA-tvmvD-N-tev-C	This paper	N/A
pSCD-GSDMA-tevD-tvmv-N	This paper	N/A
pSCD-GSDMA-tvmvD-N	This paper	N/A
pSCD-nTEVP	This paper	N/A
pSCD-cTEVP	This paper	N/A
pSCD-nTEVP-RBD	This paper	N/A
pSCD-cTEVP-RBD	This paper	N/A
PiggyBac Transposase Expression Vector	System Biosciences	Cat# PB210PA-1
pSCD-PB-HRAS-G12V-2A-Cerulean	This paper	N/A
pSCD-LV-Citrine-tev-Deg	This paper	N/A
M-pSPAX2	This paper	N/A
pSCD-LV-GSDMA-N-tev-C	This paper	N/A
pSCD-LV-Casp3-L-tev-S	This paper	N/A
pSCD-LV-Casp3-L-tev-S-CAAX	This paper	N/A
pSCD-LV-TEVP-CAAX	This paper	N/A
pSCD-GSDMA-Z-C	This paper	N/A
pSCD-LV-GSDMA-Z-C	This paper	N/A
pSCD-GSDMA-N-Z	This paper	N/A
pSCD-LV-GSDMA-N-Z	This paper	N/A
Deposited data		
Raw and processed data	This paper	CaltechDATA link: https://data.caltech.edu/communities/synpoptosis
Software and algorithms		
ImageJ	NIH	https://imagej.net/nih-image/
SnapGene 4.0.8	Dotmatics	https://www.snapgene.com/
PyMol (v. 2.5.3)	Schrödinger	https://www.schrodinger.com/products/pymol
FlowJo (v. 10.8.1)	BD Biosciences	https://www.flowjo.com/
GraphPad Prism 9	Graphpad	https://www.graphpad.com/scientific-software/prism/
Custom data analysis codes	This paper	GitHub link: https://github.com/Shiyu-Xia/synpoptosis
Other		
Multiplate 96-well PCR plates	Bio-Rad	Cat# ML9601
PCR strip tubes with flat caps	Bio-Rad	Cat# TCS0803
1.7-ml Eppendorf tubes	Fisher Scientific	Cat# 14-222-168
15-ml conical centrifuge tubes	Fisher Scientific	Cat# 14-959-70C
50-ml Falcon conical tubes	Fisher Scientific	Cat# 14-959-49A
Petri dishes	Fisher Scientific	Cat# FB0875713
Sterile basins	Fisher Scientific	Cat# 13-681-501
100-mm cell-culture dishes	Fisher Scientific	Cat# 12-567-650
40-μm cell strainers	Fisher Scientific	Cat# 08-771-1
96-well round-bottom flow cytometry plates	Fisher Scientific	Cat# 08-772-17
96-well cell-culture plates	Fisher Scientific	Cat# 08-772-3
24-well cell-culture plates	Fisher Scientific	Cat# 08-772-1
12-well cell-culture plates	Fisher Scientific	Cat# 08-772-29
6-well cell-culture plates	Fisher Scientific	Cat# 08-772-1B
T25 tissue-culture flasks	CELLTREAT	Cat# 229331
0.45 μm cellulose acetate syringe filter	VWR	Cat# 28145-481

## Highlights

- Synthetic synpoptosis protein circuits proteolytically control apoptosis and pyroptosis
- Induced death programs override endogenous cell preferences and allow mixed death modes
- Synpoptosis circuits can be coupled to inputs to enable cell state-specific activation
- Engineered sender cells can kill other cells through intercellular circuit transmission
