# Supplementary material for: Synthetic protein circuits for programmable control of mammalian cell death
Source: Cell. Author manuscript; Available in PMC 2024 May 26. (PMC11127782; doi:10.1016/j.cell.2024.03.031)
Supplement: 1 — Figure S1. Synpoptosis circuits orthogonally control cell death, related to Figure 1 (A) HEK cells express negligible levels of endogenous GSDMs. Expression data were obtained from the Human Protein Atlas (proteinatlas.org). (B) We performed the majority of experiments in the paper using the transient transfection method, as shown in the schematic with mock data. We co-transfected the cells with plasmid DNA encoding the synthetic circuits and a fluorescent protein marker. After 16–24 hours post-transfection, we collected both floating and attached cells for staining and flow cytometry. (C) There are several possible sources of cells that stain low for the fluorescent co-transfection marker (Cherry) and high for the death dye (Annexin). First, the transfection reagent itself causes some toxicity; second, the cell collection procedure involving pelleting and resuspending kills some cells; and third, when the circuits kill the cells, they also reduce the level of the fluorescent protein in the cells, making some transfected cells appear Cherry-low. Given the uncertain origins, we focused on Cherry-high cells by gating, which allowed us to definitively attribute the observed cell death to the transfected synthetic circuits. Data represent three independent experiments. (D) Three viral proteases, TEVP, TVMVP, and HCVP, orthogonally activated their cognate engineered GSDMs containing the cleavage sites (tev, tvmv, and hcv, respectively). Data represent three independent experiments. Colors indicate means. Figure S2. Synpoptosis circuits show typical features of natural death programs, related to Figure 2 (A) In transient DNA transfection experiments, engineered auto-inhibited caspase-3 or GSDMA induced modest cell death when they were highly expressed, without the activating TEVP. Within each bin on the x-axis, dots represent biological replicates (distinct culture wells). (B) Titrating the DNA amounts of the circuit plasmids enabled dose-dependent control over the fracti [file NIHMS1983030-supplement-1.pdf]

A **Lack of endogenous GSDMs**

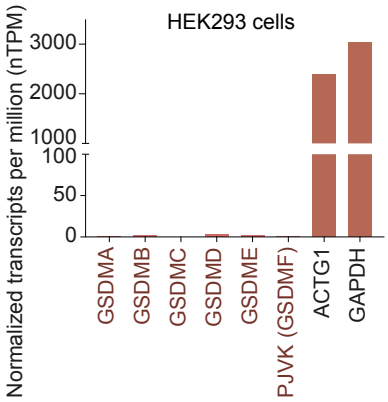

B **Characterizing synpoptosis circuits by Annexin and Sytox staining**

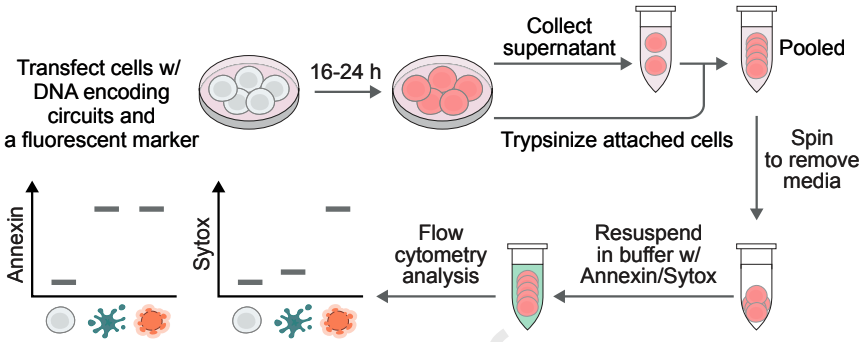

C **Directing analysis towards circuit-transfected cells**

Condition	Cell treatment	Cell analysis procedure
1	Untreated	Discard supernatant → Trypsinize attached cells → Add buffer w/ Annexin → Flow cytometry
2	Mock transfected	
3	Untreated	Collect supernatant → Trypsinize attached cells → Pool the above → Spin to remove media → Resuspend the pellet in buffer w/ Annexin → Flow cytometry
4	Mock transfected	
5	Transfected with Cherry	
6	Transfected with Cherry and an apoptosis circuit (Caspase-3 L-tev-S + TEVP)	

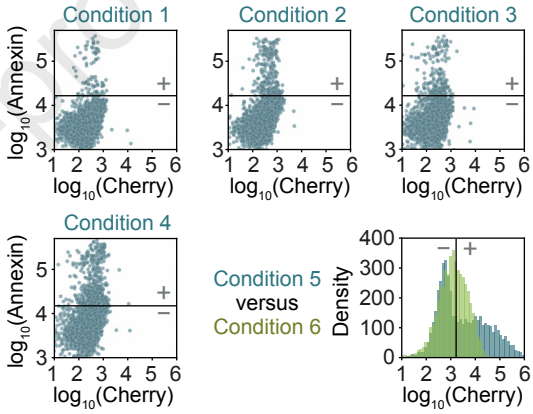

D **Orthogonal protease-executioner pairing**

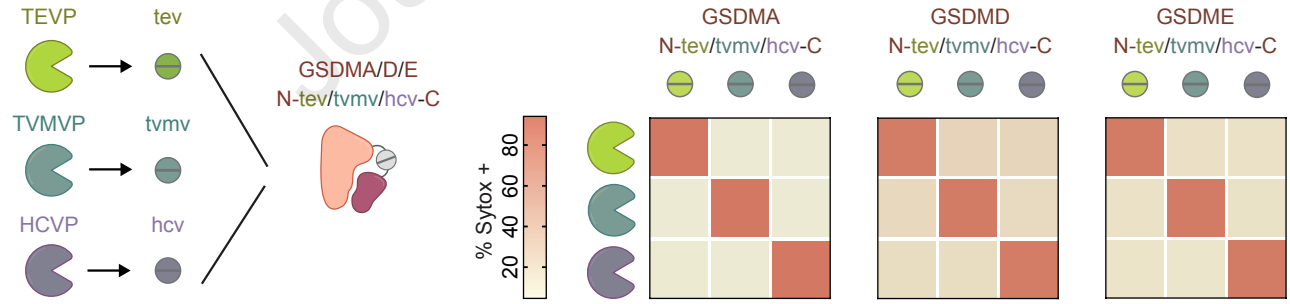

Journal Pre-proof

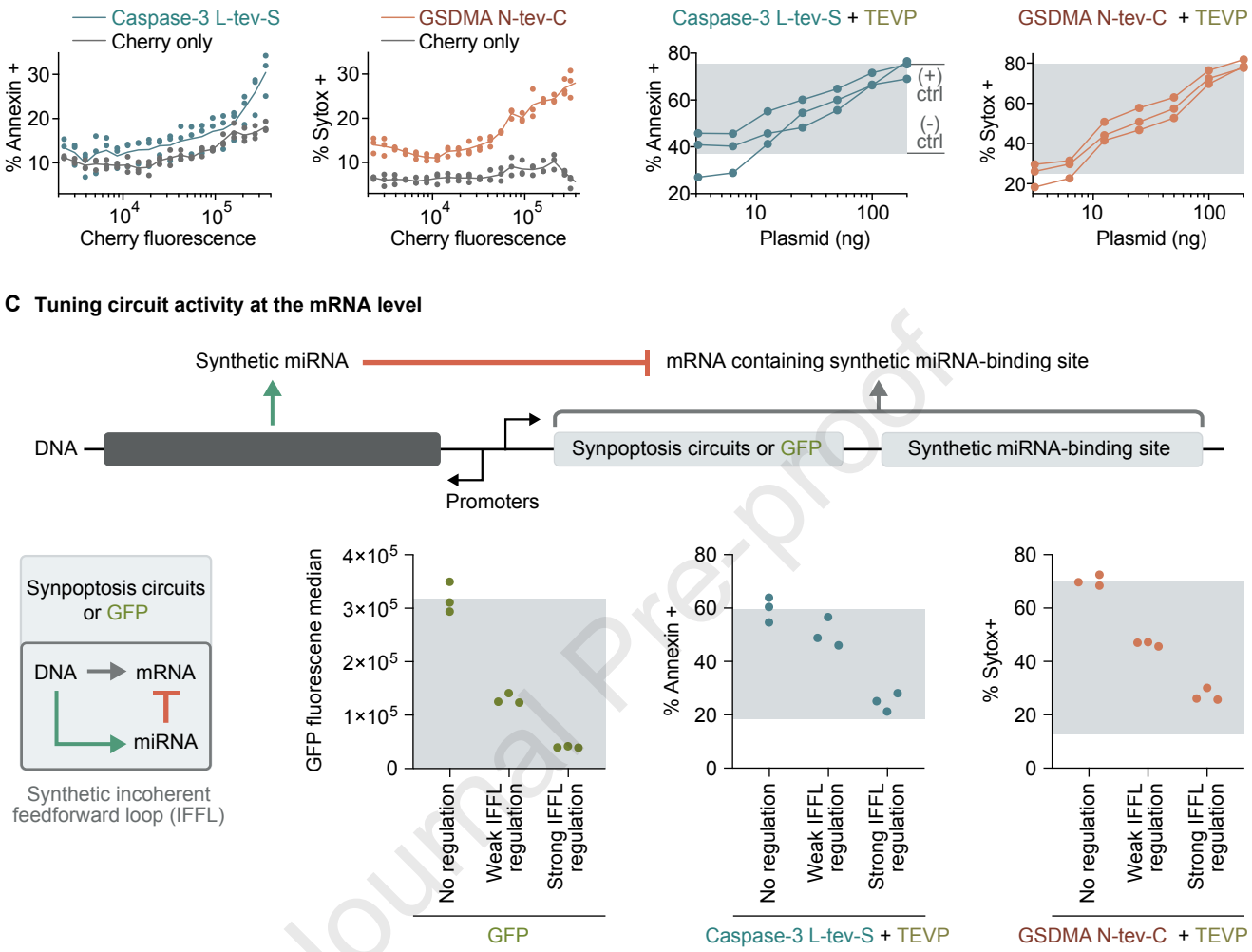

Journal Pre-proof

A **Inhibition of wildtype GSDME N by an inactive mutant**

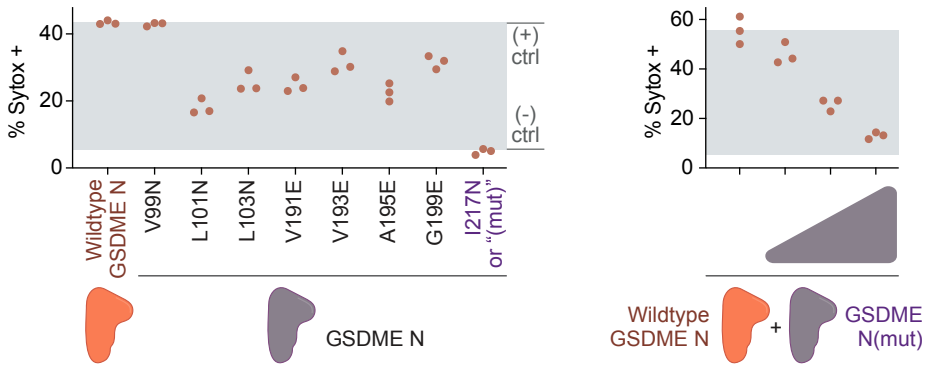

B **Promoting apoptosis to various degrees**

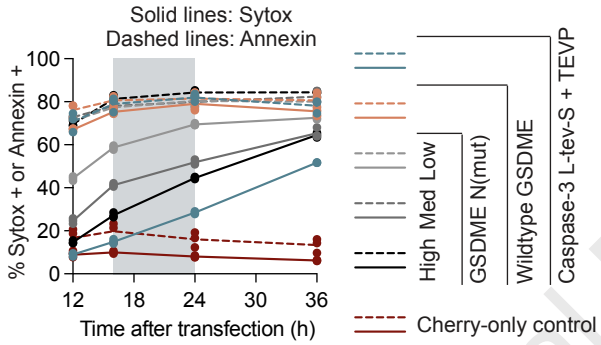

C **Independence of endogenous GSDMD**

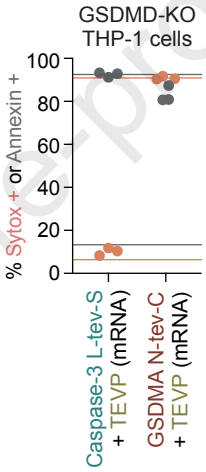

Journal Pre-proof

A More synthetic apoptosis gates

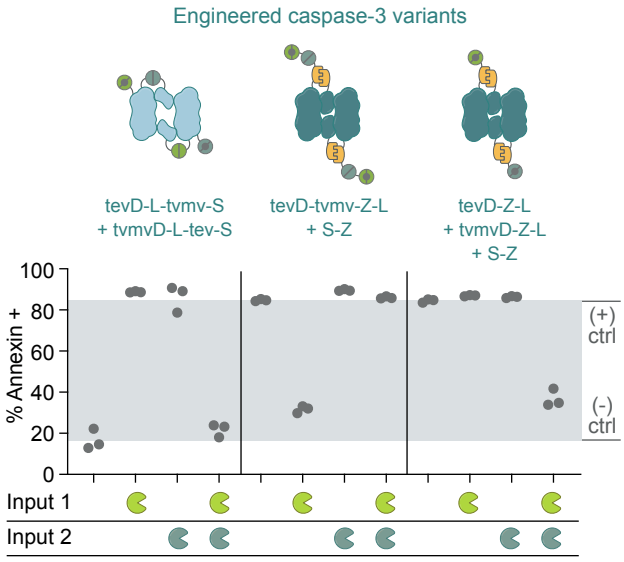

B More synthetic pyroptosis gates

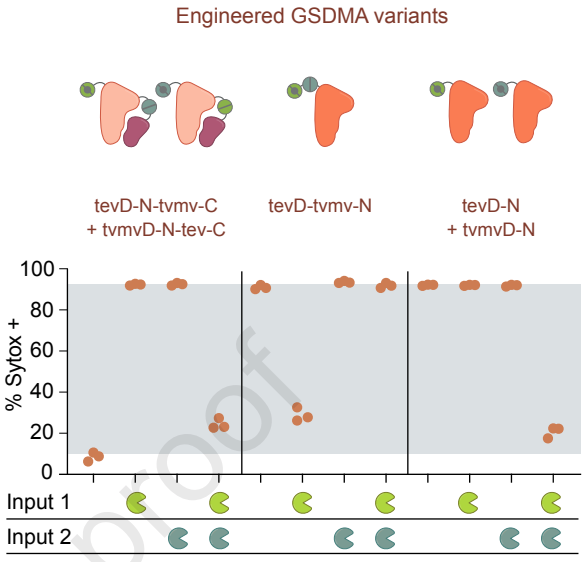

Journal Pre-proof

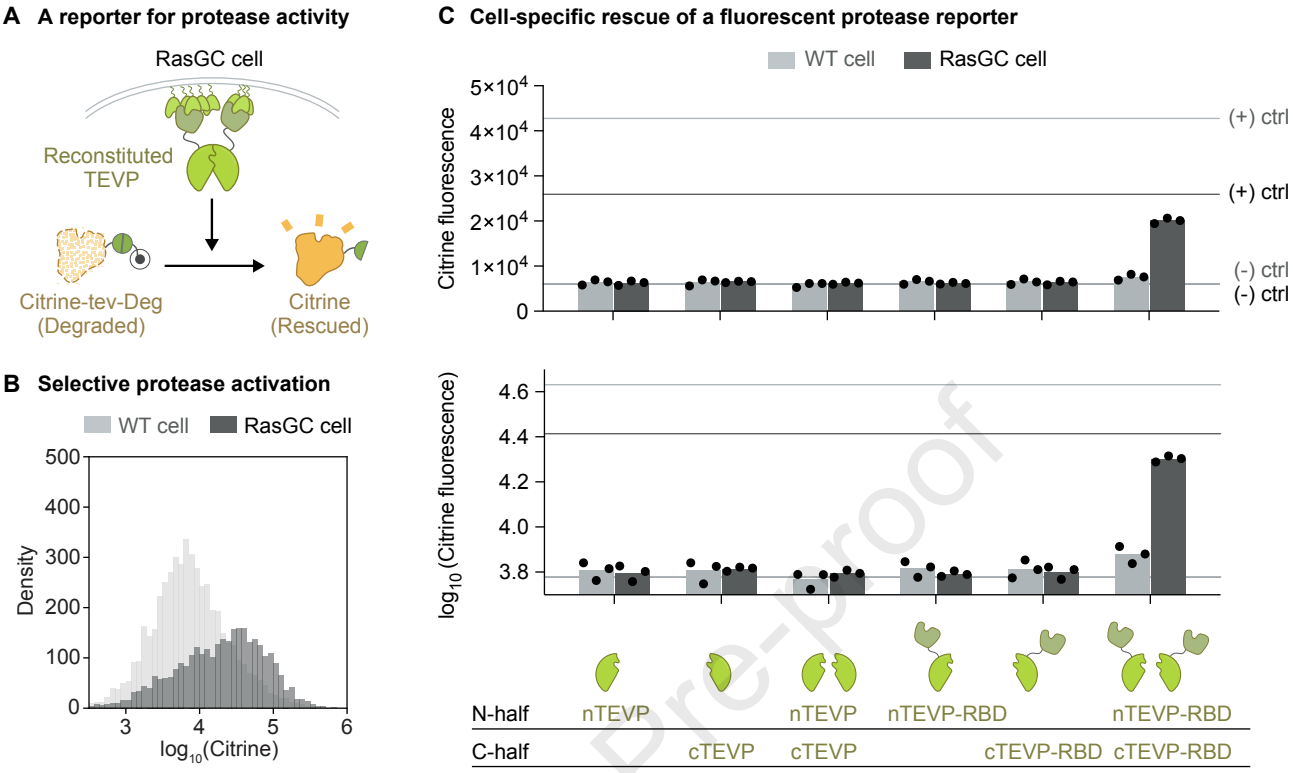

Journal Pre-proof

A Intrinsic toxicity of the split-sender system

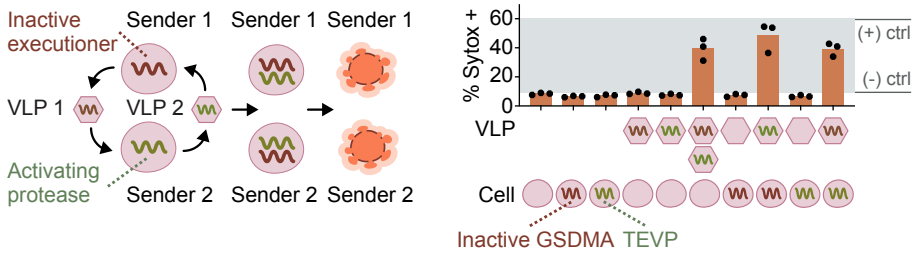

B Silencing of an active executioner

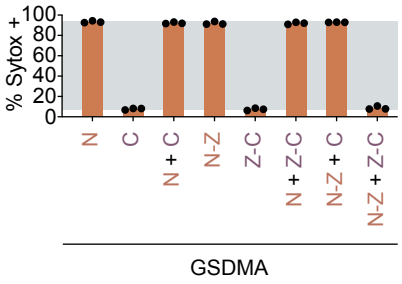

C Immunity to toxic VLPs

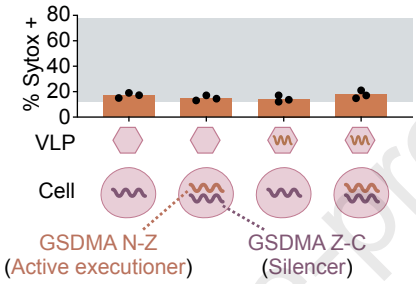

D Protection from cell death by the engineered silencer

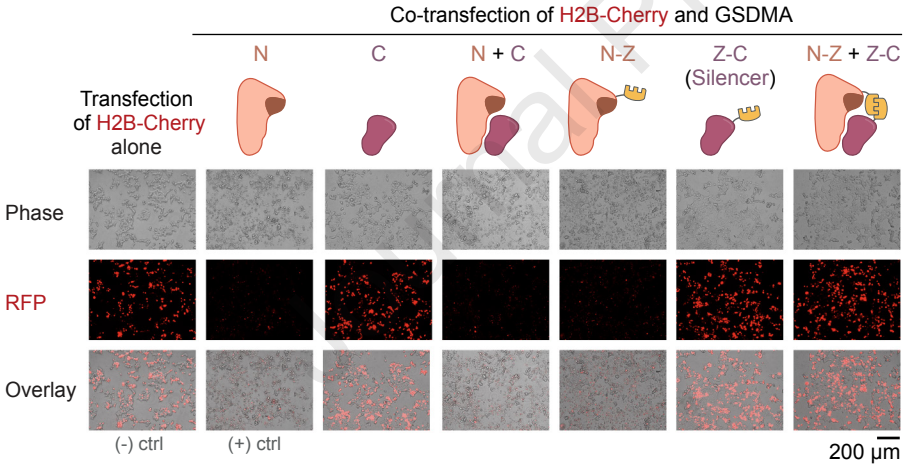

E Single-sender system in a co-culture

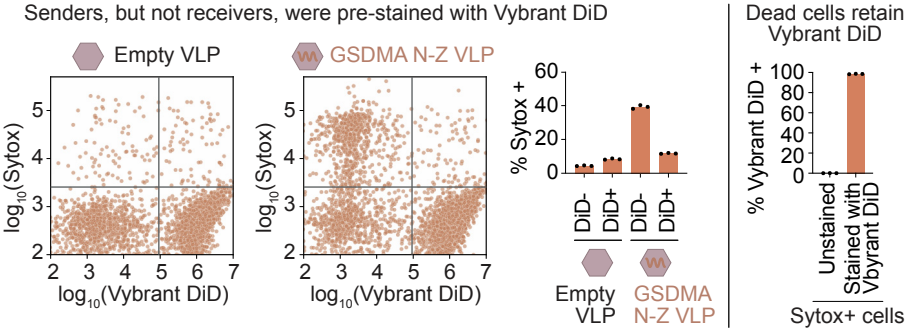

Final Preproof
